# Controlling bias and inflation in epigenome- and transcriptome-wide association studies using the empirical null distribution

**DOI:** 10.1186/s13059-016-1131-9

**Published:** 2017-01-27

**Authors:** Maarten van Iterson, Erik W. van Zwet, the BIOS Consortium, Bastiaan T. Heijmans

**Affiliations:** 1 Molecular Epidemiology section, Department of Medical Statistics and Bioinformatics, Leiden University Medical Center, Leiden, the Netherlands; 2 Department of Medical Statistics and Bioinformatics, Leiden University Medical Center, Leiden, the Netherlands

**Keywords:** Epigenome- and transcriptome-wide association studies, Bias, Inflation, Empirical null distribution, Gibbs sampler, Meta-analysis

## Abstract

We show that epigenome- and transcriptome-wide association studies (EWAS and TWAS) are prone to significant inflation and bias of test statistics, an unrecognized phenomenon introducing spurious findings if left unaddressed. Neither GWAS-based methodology nor state-of-the-art confounder adjustment methods completely remove bias and inflation. We propose a Bayesian method to control bias and inflation in EWAS and TWAS based on estimation of the empirical null distribution. Using simulations and real data, we demonstrate that our method maximizes power while properly controlling the false positive rate. We illustrate the utility of our method in large-scale EWAS and TWAS meta-analyses of age and smoking.

**Electronic supplementary material:**

The online version of this article (doi:10.1186/s13059-016-1131-9) contains supplementary material, which is available to authorized users.

## Background

The large-scale analysis of epigenome and transcriptome data in population studies is thought to answer fundamental questions about genome biology and will be instrumental in linking genetic and environmental influences to disease etiology [1, 2]. Worldwide, research groups are now joining forces to generate and analyze such data [3–7] complementary to the vast resources of genetic data that are already available and have been used successfully in genome-wide association studies (GWAS). While the analysis tool box for GWAS has matured, the development of effective methodology for the analysis of epigenome- and transcriptome-wide association studies (EWAS and TWAS) is a nascent field of research. In an EWAS, DNA methylation levels of typically hundreds of thousands of CpG dinucleotides are individually tested for association with an outcome of interest, while in a TWAS this is done for expression levels of tens of thousands of genes. Currently, EWAS and TWAS analysis heavily relies on approaches specifically designed for GWAS. However, epigenome and transcriptome data are crucially different from genetic data. They are quantitative measures (and not discrete like genotypes) that are subject to major confounding effects of technical batches and biological influences, including cellular heterogeneity [2, 8]. Furthermore, molecular phenotypes such as DNA methylation and gene expression often show stronger associations with phenotypic traits or complex diseases than genotypic markers.

A key aspect of the analysis of ome-wide association studies is the control of test-statistic inflation. Inflation of test statistics leads to an overestimation of the level of statistical significance and dramatically increases the number of false positive findings [9]. This has always been a major concern in GWAS, but inflated test statistics are also observed in EWAS [10, 11]. Often the level of inflation exceeds that observed in GWAS, yet it is generally not corrected [12]. In GWAS, test-statistic inflation is commonly addressed using genomic control in which the inflated test statistics are divided by the genomic inflation factor. The genomic inflation factor estimates the amount of inflation by comparing observed test statistics across all genetic variants to those expected under the hypothesis of no effect [9]. Recent work pointed out crucial limitations of genomic control in GWAS [13, 14]. Notably, the genomic inflation factor was shown to provide an invalid estimate of test-statistic inflation when the outcome of interest is associated with many, small genetic effects [13]. In EWAS and TWAS, this is the rule rather than the exception. Moreover, test statistics may not only be subject to inflation but also to bias [15], which is not corrected for when using genomic control. Bias of test statistics leads to a shift in the distribution of effect sizes and is driven by confounding [16, 17], a prominent feature of EWAS and TWAS but much less of a concern in GWAS [18]. Thus, this calls for the development of new methods specifically designed to address test-statistic inflation and bias in EWAS and TWAS analyses.

Although generally ignored, genomic control will overestimate the actual inflation unless it is estimated on the basis of genetic variants not associated with the outcome of interest [9, 19]. A Bayesian outlier model [20] was proposed to solve this issue; it estimates inflation while assuming a fixed and small number of 10 associated genetic variants. Although this is an improvement for GWAS with few associations, it will not be sufficient to solve the overestimation of inflation in EWAS and TWAS, which typically yield substantially more associations. Nor does it address the occurrence of test-statistic bias. In the statistical literature, alternative methods have been proposed in the context of large-scale multiple hypothesis testing where an empirical null distribution is used for inference [16, 21–23]. The utility of these approaches in EWAS and TWAS, however, remains to be evaluated.

Here, we use simulation studies and large-scale methylome (*n*=2203) and transcriptome (*n*=1910) data [24, 25] to show that correcting inflated test statistics by applying genomic control is too conservative for EWAS and TWAS and that test-statistic bias cannot be ignored. Moreover, we demonstrate that test-statistic bias and inflation are represented by the mean and standard deviation of the empirical null distribution and propose a Bayesian method for its estimation. Application of state-of-the-art batch correction methods, including *SVA* [26], *RUV* [27], and *CATE* [17], were not able to remove all test-statistic bias and inflation. Hence, the resulting test statistics require empirical calibration to achieve optimal statistical power while controlling the number of false positives at the desired level. We develop a Bayesian method for estimation of the empirical null distribution and propose a bias and inflation correction implemented as an R/Bioconductor [28, 29] package *BACON*. Finally, we show the utility of our method by performing an EWAS and TWAS meta-analysis of two commonly studied outcomes: age and smoking status.

## Results

### The genomic inflation factor is not suitable to measure inflation in EWAS/TWAS

**Fig. 1:**
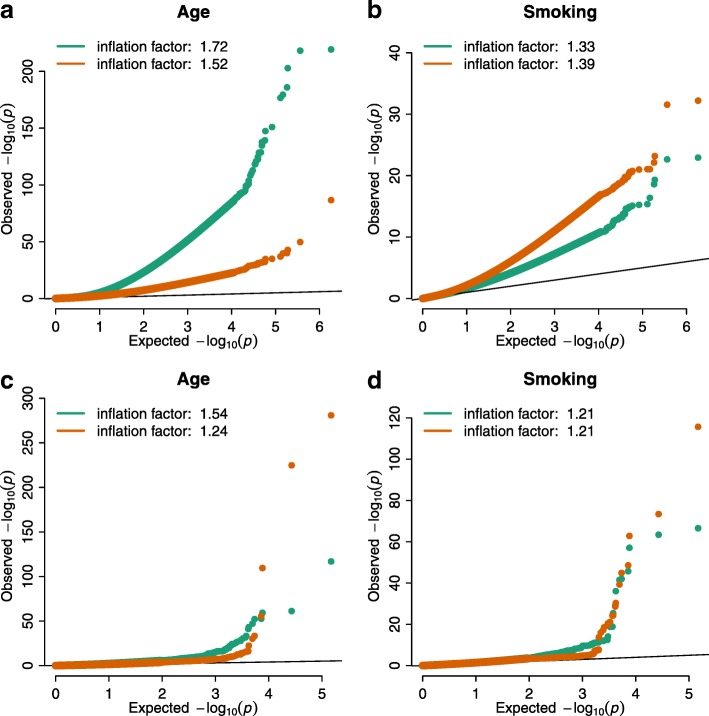
Inflated epigenome- and transcriptome-wide association studies. Quantile-quantile (*QQ*) plots for EWAS (panels **a** and **b**) and TWAS (panels **c** and **d**) performed on the LifeLines (*LL*) and Leiden Longevity Study (*LLS*) cohorts for the phenotypes age and smoking status. Results for LL are indicated in *green* and LLS in *orange*. QQ-plots show the observed minus log10-transformed *P* values obtained from a linear model corrected for known biological and technical covariates against quantiles from the theoretical null distribution. Strong inflation, as estimated according to $\protect {\lambda _{{\chi _{1}^{2}}}}$ [9], was observed for both EWAS and TWAS of age, while for the EWAS and TWAS of smoking the amount of inflation is smaller (notice different y-axis scales)

We performed an EWAS and TWAS of age and smoking status using subsets of 500 individuals from two population cohorts, namely the Leiden Longevity Study (LLS) and LifeLines (LL) (Additional file 1: Table S1). The analyses were adjusted for known technical and biological covariates (including measured white blood cell counts) within a linear model framework. Inflation of test statistics was observed in all of the eight analyses (two cohorts, two data types, and two outcomes; Fig. 1). The amount of inflation estimated using the commonly used genomic inflation factor [9] varied substantially across analyses and ranged from 1.33 to 1.72 for the EWAS and from 1.21 to 1.54 for the TWAS (Fig. 1).

However, the genomic inflation factor appeared to be correlated with the expected number of true associations. For example, the genomic inflation factor was higher for age than smoking status, and previous studies showed that age is associated with many more differentially methylated sites and differentially expressed genes than smoking status [3–7]. For the analysis of age, the genomic inflation factor was higher for LL than LLS, which can be attributed to the higher statistical power for LL (age range 21 years) than LLS (age range 9 years).

**Fig. 2:**
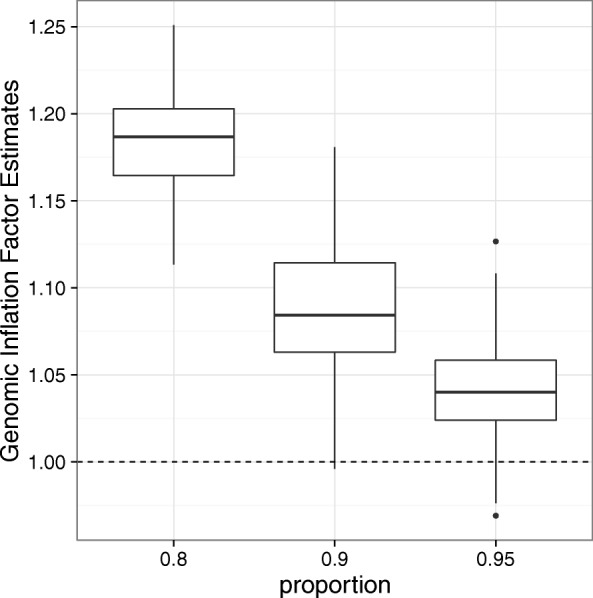
The genomic inflation factor overestimates inflation in the presence of a moderate proportion of true associations. The box-plot summarizes the estimated inflation for simulated data with different amounts of true associations. One hundred sets of test statistics were generated with different amounts of true associations (20%, 10% and 5%) but without any true inflation; i.e., the inflation factor should be equal to one. The genomic inflation factor was calculated using $\lambda _{{\chi _{1}^{2}}}$ [9]. A clear dependence on the number of true associations is seen for the genomic inflation factor

A simulation study substantiated the impression that the genomic inflation factor depends on the number of true associations (Fig. 2). In fact, this result can be derived mathematically [9]. We conclude that the genomic inflation factor commonly overestimates the true level of test-statistic inflation in EWAS and TWAS.

### EWAS/TWAS not only suffer from inflation but also from test-statistic bias

**Fig. 3:**
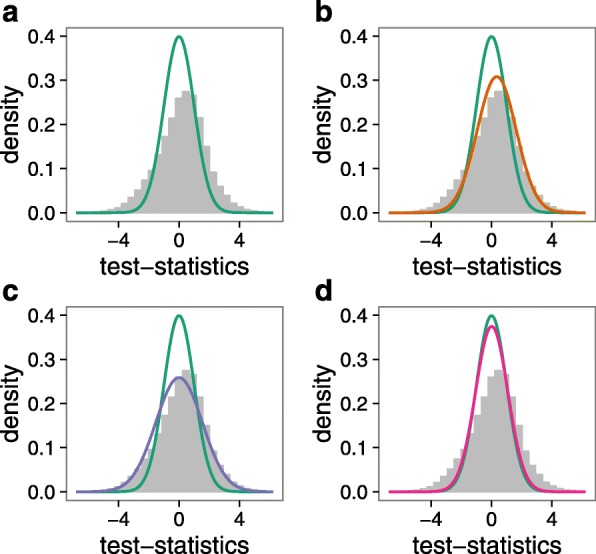
Bias in transcriptome-wide association studies. Histogram of test statistics from the TWAS of age in the LifeLines (*LL*) cohort. Each panel shows a different null distribution. **a** Theoretical null (*green*): normal distribution with mean and variance (0.0, 1.0^2^), **b** empirical null (*brown*): normal distribution with estimated mean and variance using our Bayesian method (0.23, 1.5^2^), **c** inflated null (*purple*): normal distribution with zero mean and variance equal to the estimated inflation estimated using the genomic control method (0.0, 1.5^2^), and **d** permutation null (*pink*): normal distribution with permutation-based estimates of mean and variance (−0.006, 1.1^2^). For comparison the theoretical null (*green*) is shown in each panel

While quantile-quantile plots of expected versus observed test statistics, or their corresponding *P* values, are frequently used to visualize inflation (Fig. 1), the alternative representation through a histogram of test statistics reveals a second artifact, namely a bias of the test statistics (Fig. 3 a and Additional file 1: Figure S1). This bias is visible as a deviation of the mode of the observed test statistics from zero, which is the mode of the standard normal distribution. Since the majority of features (being genetic variants, CpGs, or genes) are assumed not to be associated with the outcome of interest, test statistics obtained from a linear model should follow a standard normal distribution (i.e., centered at zero). We observed test-statistic bias in the EWAS and TWAS of age and smoking irrespective of cohort and outcome (Additional file 1: Figure S1). Genomic control does not address bias because it uses a normal distribution with the mode fixed at zero (Additional file 2). The misspecification of the observed distribution of test statistics by genomic control is illustrated in Fig. 3 c. Note that even permutation-based approaches, which are often assumed to rescue violations of assumptions regarding the theoretical null distribution, do not result in a proper null distribution, and both test-statistic bias and inflation persist [16, 30] (Fig. 3 d). We mathematically derived that unobserved confounding factors introduce bias in the analysis of high-dimensional data (Additional file 2), thus expanding on earlier work by Rao [15].

### Estimating test-statistic bias and inflation

**Fig. 4:**
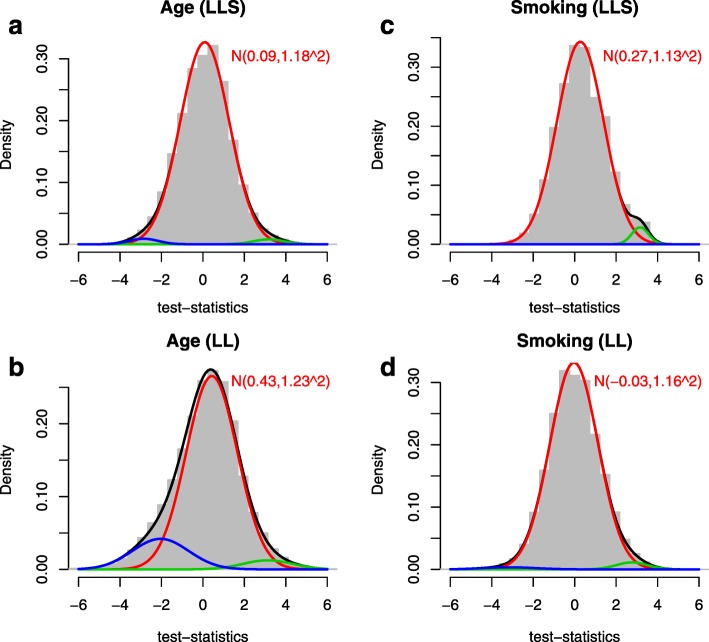
Histogram of test statistics for TWAS on age (**a** and **b**) and smoking status (**c** and **d**) performed on two cohorts: LifeLines (*LL*) and Leiden Longevity Study (*LLS*). The lines represent the three-component normal mixture fitted as estimated using our Bayesian method. The *black line* represents the fit of the mixture, the red line the fit of the null component (the empirical null distribution with estimated mean and variance reported). The *blue and green lines* represent the estimated fits of the alternative components (proportion of positively and negatively associated features)

Both bias and inflation represent deviations from the theoretical null distribution: bias (i.e., mean), a deviation from zero, and inflation (i.e., standard deviation) (Additional file 2). Hence, estimating the amount of bias and inflation is identical to estimating the parameters of the empirical null distribution. We developed a Bayesian method to estimate the empirical null distribution from an observed set of test statistics and thus simultaneously obtain estimates of bias and inflation. The method fits a three-component normal mixture to the observed set of test statistics using a Gibbs sampling algorithm [31]. One component reflects the null distribution with mean and standard deviation representing bias and inflation. The other two components with a positive and a negative mean capture the fraction of true associations observed in the data, which is assumed to be an unknown minority of tests (Fig. 3 b, Fig. 4, and Additional file 1: Figure S2). Hence, our method simultaneously provides estimates for the amount of bias and inflation without being affected by an unknown proportion of true associations (Additional file 1: Figure S3). We compared our method to alternative approaches for estimation of the empirical null distribution [16] in a simulation study. This showed that the performance of our method is equal to or better than those of the previous methods under various scenarios. Moreover, our method resulted in the most stable estimation of the inflation, which suggests that other methods randomly over- or underestimate the level of inflation (Additional file 1: Figure S4 and Additional file 3).

### Correction for unobserved covariates reduces test-statistic bias and inflation

The primary causes of inflation and bias are thought to be unmeasured technical and biological confounding [8, 16], e.g., population substructure, batch effects, and cellular heterogeneity. Various methods have been developed to reduce the impact of these unmeasured factors in high-dimensional data [17, 26, 27, 32–34]. We applied six of these methods to adjust an EWAS and TWAS of age in 500 individuals, a subset of the LLS cohort, and investigated their impact on test-statistic bias and inflation. All approaches reduced the amount of bias and inflation as compared with a model using known covariates only (Table 1 and Additional file 1: Table S2). Nevertheless, residual bias and inflation were observed. Therefore, we designed a two-stage method in order to preserve statistical power while appropriately controlling the number of false positives. First, we performed an analysis that corrects for known biological and technical covariates plus estimated unobserved covariates, followed by estimating and adjusting the residual bias and inflation using the empirical null distribution. In the adjustment step, *P* values are calculated using the empirical null distribution instead of the standard normal or the inflated normal that is used by the genomic control method. A complication of the genomic inflation factor is that it estimates the variance of the null distribution ($\lambda _{{\chi ^{2}_{1}}}$), whereas the standard deviation ($\sqrt {\lambda _{{\chi ^{2}_{1}}}}$) is required for genomic control on normally distributed test statistics resulting from linear models with a continuous outcome (here, DNA methylation and gene expression data). Furthermore, it is important to note that bias not only results in incorrect test statistics and *P* values but also results in biased estimates of effect sizes. To evaluate the performance of the two-stage method, we conducted a numerical simulation. To account for unmeasured confounding, we selected *CATE*, a state-of-the-art method that was shown to have superior performance in estimating unobserved covariates as compared with alternative methods [17]. Our Bayesian method in combination with *CATE* yielded the highest power with the fraction of false positives close to the nominal level (0.058±0.0052). In contrast, methods that ignore unobserved covariates led to high false positive rates and methods that use genomic control resulted in low power (Table 2). Also, the test-statistic calibration that has been proposed to use in combination with *CATE* [17] was conservative, resulting in low power, which is in line with the fact that this method is closely related to genomic control.

**Table 1:** Correction for unobserved covariates reduces test-statistic bias and inflation

Method	Genomic infl. factor	Bayesian infl. factor (bias)
	$\sqrt {\lambda _{{\chi _{1}^{2}}}}$	
1. No	1.322	1.229 (0.000)
2. Known	1.237	1.169 (0.080)
3. PC (1)	1.257	1.183 (0.048)
4. PC (2)	1.222	1.147 (-0.002)
5. PC (3)	1.160	1.090 (-0.139)
6. SVA (3)	1.181	1.116 (0.022)
7. RUV-Res (3)	1.332	1.166 (0.086)
8. RUV-Emp (3)	1.197	1.130 (0.021)
9. CATE (2)	1.161	1.077 (0.053)

Genomic inflation factor estimates ($\sqrt {\lambda _{{\chi _{1}^{2}}}}$, square root since the test statistics follow a normal distribution and not a *χ* ^2^) and inflation factor (and bias) estimates obtained using the Bayesian estimation of the empirical null distribution from test statistics obtained by fitting linear models for a TWAS of age in the Leiden Longevity Study (*LLS*) cohort subset of 500 individuals. Nine different models were fitted using different approaches to estimate and correct for unobserved covariates: (1) only known covariates, (2) including known covariates, (3), (4), and (5) known covariates plus one, two, or three principal component(s), respectively, (6) known covariates plus three optimal surrogate variables estimated using *SVA* [26], (7) known covariates plus three unobserved covariates estimated using *RUV* [32] with the residual method, (8) known covariates plus three unobserved covariates estimated using *RUV* [32] with the empirical method, (9) known covariates plus two optimal latent variables estimated using *CATE* [17] (within parentheses the number of principal components, optimal number of surrogate variables, or optimal number of latent factors)

**Table 2:** Bias and inflation correction after adjustment for confounding factors yields optimal power

Method	False positive rate	Power
	mean (stdev)	mean (stdev)
	No confounding adjustment	
No correction	0.720 (0.0360)	0.720 (0.049)
Genomic control	0.001 (0.0020)	0.005 (0.007)
Bayesian control	0.029 (0.0076)	0.050 (0.018)
	Confounding adjustment	
No correction	0.060 (0.0056)	0.860 (0.037)
Calibration	0.030 (0.0042)	0.770 (0.053)
Bayesian control	0.058 (0.0052)	0.860 (0.041)
oracle	0.052 (0.0052)	0.850 (0.039)

Mean and standard deviation of the number of false positives and true positives (power) for a simulation study repeated 100×. Data were generated according to the simulation setup of Wang et al. [17]. The table summarizes the results for the naive approach of no adjustment for confounding factors and adjusting for confounding factors using *CATE*. Both in combination with different approaches are used to control for inflation (and bias): no correction, correction using genomic control, correction using the median and median absolute deviation (*MAD*), calibration [17], and using our Bayesian method. As a comparison the oracle method is shown where the simulated confounding factors have been added to the linear model

In addition to confounding, correlation between features (i.e., CpGs and genes) may cause test-statistics inflation or bias. A second simulation study showed that if test statistics are correlated, our Bayesian method properly controls the false positive rate while preserving power (Table 3). Again, the application of genomic control is too conservative (Table 3 and Additional file 3).

**Table 3:** Empirical null estimates from correlated test statistics yield proper control of the false positives rate without any reduction in power

Method	False positive rate	Power
	mean (stdev)	mean (stdev)
	Uncorrelated	
No correction	0.050 (0.003)	0.770 (0.020)
Genomic control	0.028 (0.003)	0.710 (0.020)
Bayesian control	0.052 (0.003)	0.770 (0.020)
	Correlated	
No correction	0.040 (0.030)	0.770 (0.020)
Genomic control	0.023 (0.006)	0.730 (0.090)
Bayesian control	0.054 (0.020)	0.800 (0.060)

Mean and standard deviation of the number of false positives and true positives (power) for a simulation study repeated 100×. Correlated test statistics were generated according to the simulation setup of Efron [51]. The table summarizes the results for uncorrelated test statistics and correlated test statistics, without any correction for inflation or bias, using genomic control and using our Bayesian method

### Fixed-effect meta-analysis with control for bias and inflation

**Fig. 5:**
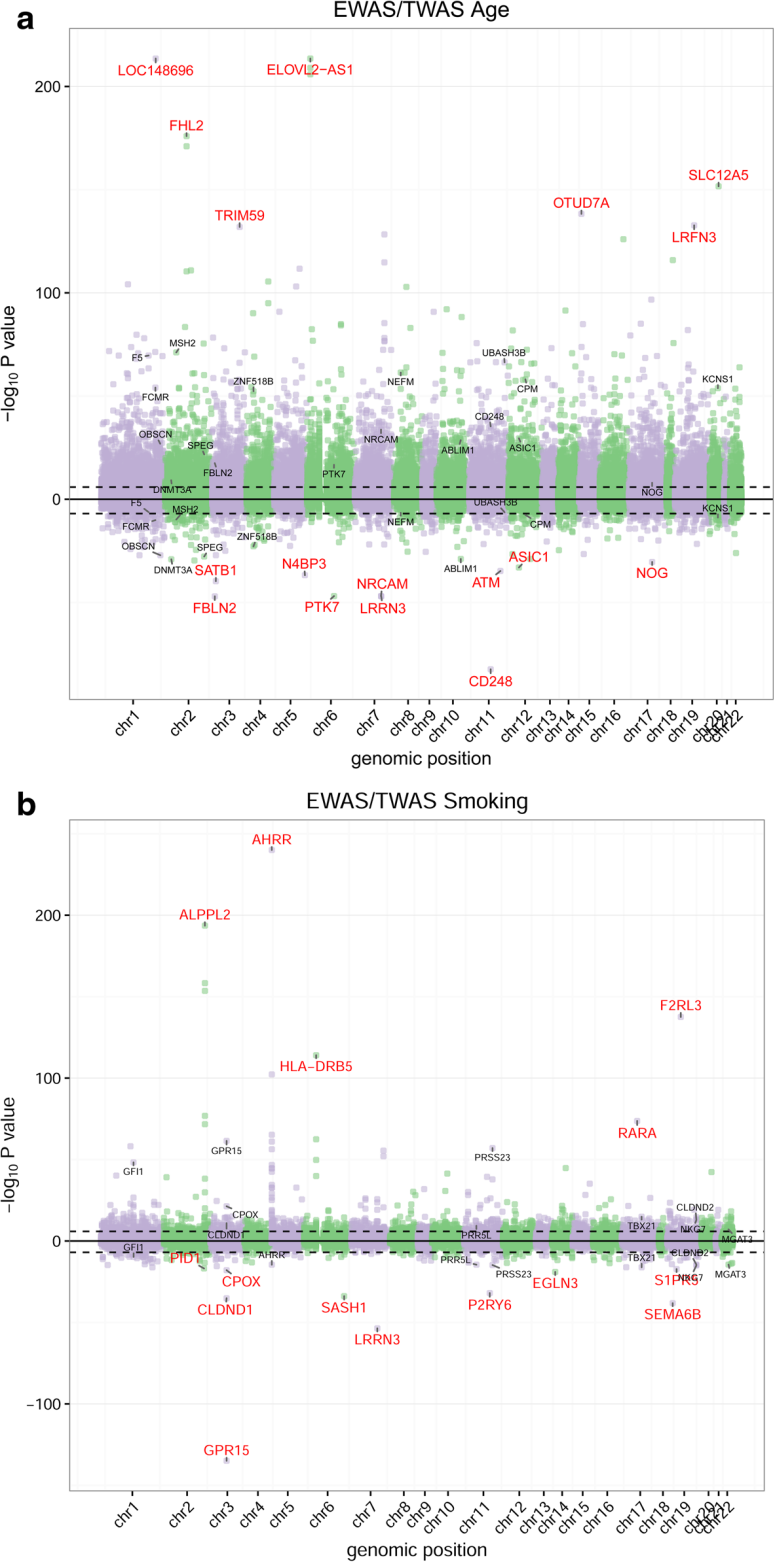
Manhattan plots meta-analyses across four cohorts of EWAS and TWAS of age and smoking status. Panel **a** shows the meta-analysis results of the EWAS of age as − log10*P* values and with reverse sign for the TWAS of age as log10*P* values. Panel **b** shows the same figure for smoking. The *black line* indicates 0.05 Bonferroni thresholds. *Red gene names* highlight the top 10 (nearest) genes resulting from the EWAS and TWAS. *Black gene names* denote genes that were identified in both the EWAS and TWAS (genes for EWAS are the genes closest to the significant CpG)

A main development in the field of EWAS and TWAS, analogous to current practice in GWAS, is the combined analysis of multiple population studies to detect an increasing number of associations including those with small effect sizes. Fixed-effect meta-analysis combines estimated effect sizes and their standard errors from different studies to construct pooled estimates resulting in higher precision of effect-size estimates and hence superior statistical power [35, 36]. We performed an EWAS and TWAS of age and smoking status in four cohorts totaling 2203 individuals with methylome and 1910 individuals with transcriptome data, respectively. We combined the results from the four cohorts through fixed-effect meta-analysis (Table 4 and Additional file 1: Figure S5). As observed earlier, bias and inflation remained present after addressing unmeasured confounding using *CATE*. Also estimates of inflation using genomic control were both higher and considerably more variable across analyses and cohorts than the estimates obtained using our Bayesian method (Table 4). The Bayesian method fully removed all bias and inflation. Critically, bias (< |0.03|) and inflation (< 1.14) remained minimal in the meta-analysis as compared with a meta-analysis using genomic control (Table 4). The latter contrasts to approaches in which inflation is not addressed at all or those using genomic control: both can result in high levels of inflation and bias in the meta-analysis that often are considerably higher than in the individual cohorts. The top hits identified for age and smoking included those consistently reported in previous studies [3–7]. Furthermore, the simultaneous performance of an EWAS and TWAS in a large-scale meta-analysis showed a remarkable overlap in results between the two study types of 410 and 57 genes for age and smoking, respectively (assigning the nearest gene to a CpG site) (Additional files 4–7: Tables S3a-d). For example, both DNA methylation near and expression of *CD248*, *DNMT3A*, and *FBLN2* were associated with age (Fig. 5 a), while the same was true for *GPR15*, *AHRR* and *CLDND1* for smoking (Fig. 5 b). In total 15,967 (3.5*%*) CpG sites and 1020 (2.7*%*) genes were significantly associated with age (Bonferroni-corrected *P* values < 0.05). For smoking, the number of associated CpGs and genes were 1128 (0.25*%*) and 301 (0.80*%*), respectively.

**Table 4:** Bias and inflation of test statistics for EWAS and TWAS across four cohorts on age and smoking status

		EWAS		TWAS	
		Age	Smoking	Age	Smoking
		infl. bias $\left (\sqrt {\lambda _{{\chi ^{2}_{1}}}}\right)$	infl. bias $\left (\sqrt {\lambda _{{\chi ^{2}_{1}}}}\right)$	infl. bias $\left (\sqrt {\lambda _{{\chi ^{2}_{1}}}}\right)$	infl. bias $\left (\sqrt {\lambda _{{\chi ^{2}_{1}}}}\right)$
Uncorrected	CODAM	1.17 0.100 (1.19)	1.02 0.040 (1.03)	1.13 -0.030 (1.20)	1.05 0.100 (1.06)
	LL	1.45 -0.500 (1.94)	1.07 0.009 (1.08)	1.17 0.040 (1.39)	1.15 0.080 (1.22)
	LLS	1.30 0.100 (1.36)	1.05 -0.200 (1.08)	1.18 0.050 (1.26)	1.15 -0.010 (1.17)
	RS	1.34 0.700 (1.57)	0.99 -0.100 (1.01)	1.11 -0.005 (1.12)	1.10 -0.010 (1.12)
Corrected	CODAM	1.01 -0.000 (1.01)	1.00 0.000 (1.01)	1.02 -0.010 (1.06)	1.00 0.000 (1.00)
	LL	1.00 -0.000 (1.27)	1.00 0.000 (1.01)	1.02 0.010 (1.19)	1.02 0.010 (1.06)
	LLS	1.02 0.007 (1.05)	1.00 -0.003 (1.01)	1.03 0.001 (1.07)	1.02 -0.010 (1.02)
	RS	1.00 0.000 (1.02)	0.99 0.000 (1.01)	1.02 -0.006 (1.01)	1.01 0.001 (1.02)
	1.19 -0.030 (1.47)	1.05 0.020 (1.10)	1.04 0.030 (1.28)	1.06 -0.002 (1.14)	
meta-analysis					

The table shows the bias and inflation as obtained using Bayesian method to estimate the empirical null and (within parentheses) using the genomic inflation factor $\left (\sqrt {\lambda _{{\chi ^{2}_{1}}}}\right)$ both before correction and after correction for inflation (and bias in case of empirical control). The estimated inflation for the meta-analysis results are after control for inflation and bias in the individual cohorts and (within parentheses) inflation after applying genomic control. Sample sizes of the cohorts for EWAS/TWAS were n =164/181 (*CODAM*), n =744/605 (*LL*), n =683/589 (*LLS*), and n =612/535 (*RS*)

We implemented our Bayesian method as an R/Bioconductor [28, 29] package *BACON*. *BACON* provides valid estimates of bias and inflation in large-scale analyses including EWAS and TWAS, yields corrected test statistics, and supports the streamlined application of the method to fixed-effect meta-analyses.

## Discussion and conclusion

We describe a novel Bayesian method to detect and correct for bias and inflation in epigenome- and transcriptome-wide association studies. Our method has the crucial characteristic that it is largely independent of the fraction of true associations in the data. We showed that the application of genomic control results in spurious associations because it does not address bias and, moreover, reduces power because it is sensitive to the number of true associations and thus commonly overestimates the levels of inflation. The performance of our method towards estimating the empirical null distribution of test statistics outperforms existing methods [16] by taking advantage of prior knowledge of the distribution and the composition of test statistics.

Methods that try to estimate unmeasured covariates [17, 26, 27] and those that try to recover the empirical null distribution [16] rely on the same principle. They extract information from features that are assumed not to be associated with the outcome of interest. Methods to estimate unknown covariates (e.g., *RUV*, *SVA*, and *CATE* as we used here) either use negative controls or assume the number of associated features to be sparse and, interestingly, they can be unified in a single mathematical framework [17]. Genomic control [9] yields a valid estimate of the inflation factor when calculated from features that are known not to be associated with the phenotype of interest. Similarly, the estimation of the empirical null distribution requires that the vast majority of features follow the null distribution [16]. Our Bayesian method is designed to be flexible in dealing with larger fractions of true associations, which turns out to be crucial in particular for EWAS and TWAS meta-analyses.

Our work extends the work of Devlin and Roeder [9], who originally propose to use genomic control to tackle test-statistic inflation for GWAS, and links their method to the pioneering work of Efron [16] on estimating an empirical null distribution for high-dimensional data inference. Hence, although specifically applied to EWAS and TWAS, our statistical method may have implications for any field focusing on statistical inference for high-dimensional data, whether it be omics types or imaging data.

Our method of estimating bias and inflation may resolve a common inconsistency in the current analysis of EWAS and TWAS. While it is becoming the norm to report inflation factors calculated using the traditional genomic control approach, inflation is rarely actually dealt with in the analysis, presumably because this is deemed to be too conservative. However, inflation may be substantial, in particular in a meta-analysis, and current practice is bound to introduce false positive findings. We show that estimating the inflation factor using the genomic inflation factor results both in an overestimation of the actual inflation (i.e., it is indeed conservative) and in imprecise estimates contributing to the previously unexplained, high variability across studies. Our method provides a realistic estimate of inflation that does not suffer from a high variability. Moreover, our method is the first to address the previously unrecognized issue of bias in test statistics. In conclusion, our method optimally reduces the number of false positive findings while preserving statistical power and can be seamlessly incorporated into existing work-flows for the analysis of EWAS, TWAS, and other omics data.

## Methods

### Data sets

DNA methylation data and RNA-seq data were generated within the Biobank-based Integrative Omics Studies Consortium (http://wiki.bbmri.nl/wiki/BIOS_start-). The data comprise four biobanks: Cohort on Diabetes and Atherosclerosis Maastricht (CODAM, n ≈180) [37], LifeLines (LL, n ≈700) [38], the Leiden Longevity Study (LLS, n ≈600) [39], and the Rotterdam Study (RS, n ≈600) [40]. Sample identity of DNA methylation and gene expression data was confirmed using genotype data. Both RNA-seq fastq files and DNA methylation idat files are available from the European Genome-phenome Archive (EGA) under accession number [EGA:EGAC00001000277] together with phenotypes and measured cell counts used in these analyses. Data were generated by the Human Genotyping facility (HugeF) of ErasmusMC, the Netherlands (www.glimDNA.org).

### RNA-seq data preprocessing

A detailed description of the RNA-seq data processing can be found in Zhernakova et al. [24]. Briefly, total RNA from whole blood, depleted of globin transcripts, was sequenced (2×50-bp) using the Illumina HiSeq 2000 platform, and read alignment was performed using STAR (v2.3.0). Subsequently, RNA-seq counts were normalized using *TMM* [41] and transformed to log2 counts per million. Genes that yielded zero counts for all samples across cohorts were removed, which resulted in 45,867 genes (ENSEMBLv73). For all analyses, genes with the lowest overall variance were excluded (5*%* lowest).

### 450K DNA methylation data preprocessing

The generation of genome-wide DNA methylation data is described by Bonder et al. [25]. Briefly, 500 ng of genomic DNA was bisulfite modified using the EZ DNA Methylation kit (Zymo Research, Irvine, CA, USA) and hybridized on Illumina 450K arrays according to the manufacturer’s protocols. The original idat files were generated by the Illumina iScan BeadChip scanner. Subsequently, sample quality control was performed using *MethylAid* [42]. Ambiguously mapped probes [43], probes with a high detection *P* value (> 0.01), probes with a low bead count (< 3 beads), and probes with a low success rate (missing in > 95*%* of the samples) were set to missing. Samples containing an excess of missing probes (> 5*%*) were excluded from the analysis. Subsequently, per cohort, imputation [44] was performed to impute the missing values. Functional normalization [45], as implemented in the *minfi* package [46], was used per cohort. All analyses were performed on M values. Detailed description of the 450K DNA methylation preprocessing steps are available from the git-repo Leiden450K [47].

### White blood cell count prediction

White blood cell counts (WBC), i.e., neutrophils, lymphocytes, monocytes, eosinophils, and basophils, were measured by the standard WBC differential as part of the complete blood count (CBC). A minority of samples were lacking CBC measurements. Since DNA methylation levels are informative of the white blood cell composition [48], we build a linear predictor to infer the white blood cell composition of those samples lacking WBC measurements (Additional file 2). Predicted cell counts were used in the meta-analysis. For the analyses of the cohort subsets, individuals with measured cell counts were selected.

### Association analyses

All association analyses were performed using *limma*’s lmFit function [49]. Since the sample sizes of our data were all above > 100, the empirical Bayes step was skipped. T test statistics were transformed to *P* values using a standard normal distribution. For the analysis of RNA-seq data, we first applied a *voom*-transformation [50] on the *TMM*-normalized counts while controlling for known covariates including age, gender, smoking status measured cell counts, and a technical covariate introducing a batch effect (the flow-cell identifier of the sequencing machine). For the analysis of DNA methylation data, the functional normalized beta-values [45] were transformed to M values, and again lmFit was used to obtain test statistics for the covariate of interest. Here we included age, gender, smoking status, measured cell counts, and array position as known covariates.

### Genomic control and the genomic inflation factor

The genomic inflation factor as originally proposed by Devlin and Roeder [9] is the ratio of the median of a set of trend-test statistics (i.e., obtained by the Armitage’s trend test that follows under the null hypothesis of no association a *χ* ^2^-distribution with one degree of freedom) divided by the theoretical median, . For example, let *w*_1_,*w*_2_,⋯,*w*_*p*_ be a set of *p* test statistics, following a ${\chi _{1}^{2}}$-distribution with one degree of freedom; the following estimator was proposed to quantify the amount of inflation:

1 $$ \lambda_{{\chi_{1}^{2}}} = \frac{\text{median}\{w_{1}, w_{2}, \cdots, w_{p}\}}{0.456}.  $$

Furthermore, it was proposed to control the inflated test statistics by dividing the test statistics by the estimated amount of inflation; this approach is referred to as *genomic control* [9].

In EWAS/TWAS test statistics are usually obtained from inference on the coefficients of linear regression models (instead of a trend test), i.e., *t*-test statistics that can be assumed approximately to follow a standard normal distribution (instead of a *χ* ^2^-distribution). Therefore, applying genomic control to these test statistics entails dividing by the square root of the genomic inflation factor, $\sqrt {\lambda _{{\chi ^{2}_{1}}}}$ (instead of $\lambda _{{\chi ^{2}_{1}}}$).

### Estimation of the unobserved covariates

To investigate whether adding estimated unobserved covariates reduces bias and inflation, we performed EWAS/TWAS with (1) only the covariate of interest, (2) known covariates (e.g., white blood cell counts), and either (3) known covariates plus one, (4) plus two, or (5) plus three principal components estimated from the data, and (6) known covariates with estimated unobserved covariates using *CATE* [17]. For TWAS, we additionally used *RUV* [27, 32] and *SVA* [26]. For EWAS, *iSVA* [33] and *RUVm* [34] were used. All algorithms were used with default parameters except for *CATE*, which was run using calibrate=FALSE.

### Simulation studies

#### The impact of true association on the genomic inflation factor

One hundred sets of 2000 test statistics were generated from a normal mixture distribution with different mixture coefficients (0.8, 0.90, and 0.95). The majority of the null test statistics were drawn from a standard normal, *N*(0,1), while the alternative test statistics were drawn from a normal distribution, *N*(*μ*,1), with *μ*∼*N*(0,3). An equal number of positive and negative associations were simulated. For each set of test statistics, inflation factors were calculated to investigate the impact of the number of true associations (Additional file 3). Additional file 3 shows the performance and robustness of *BACON* in estimating the empirical null distribution when different data generating approaches are used.

#### Comparing different methods that estimate the empirical null distribution

Efron proposed two methods for estimation of the empirical null distribution from a set of test statistics [16]. In order to compare the performance of those methods with our Bayesian method, sets of test statistics were generated, similar to the approach described above, but under different scenarios: scenario “equal” with equal proportion of positive and negative associations (0.05, 0.05), scenario “skewed” with only positive associations (prop. 0.1), scenario “small” similar to scenario equal with only 0.01 proportion of true associations, and scenario “close” where the distribution for the means had expected value of 1 (instead of 3). For each scenario, 2000 test statistics were generated 100 times. To estimate the empirical null distributions as proposed by Efron, we used the *locfdr* *R* package. For both methods, maximum likelihood and moment matching, default parameter settings were used (Additional file 3).

#### Simulation with unobserved confounding factors

We used the simulation setup of Wang et al. [17] to generate data with confounding factors. Briefly, data *Y*_*n*×*p*_ for *n*=100 samples and *p*=2000 features were generated according to the following model: *Y*_*n*×*p*_=*X*_*n*×1_ *β* ^*T*^+*Z*_*n*×*r*_ *γ* ^*T*^+*E*, where *Z*, represents the *r*=5 unobserved confounding factors model as *Z*|*X*=*X* *α* ^*T*^+*D*, with *α* representing the strength of confounding. Furthermore, a continuous covariate of interest, *X*, was sampled from the normal distribution. Effects were introduced by fixing 90% of the *β*s at zero while the remaining were different from zero. Both *E* and *D* represent Gaussian noise. A detailed description of the simulation setup is given by Wang et al. and is available as an *R* function *gen.sim.dat* from the package *CATE* (Additional file 3: section 5).

#### Simulation with correlated test statistics

Correlated test statistics were generated according to the approach of Efron [51] introducing a block-correlation structure among test statistics. The uncorrelated test statistics with effects generated from the normal mixture were added to the test statistics with block-correlation structure (Additional file 3: section 3.3 and section 5). The same number of repeated simulations, 100, number of test statistics, 2000, and proportion of null features, 0.9 were used.

#### The Gibbs sampler

We assume the observed set of test statistics can be modeled by a three-component normal mixture:

2 $$ f\left(x; \boldsymbol{\epsilon}, \boldsymbol{\mu}, \boldsymbol{\sigma}\right) = \sum_{j=1}^{3}\epsilon_{j}\phi\left(x;\mu_{j}, \sigma_{j}\right),  $$

with 9−1 parameters (the mixture proportions are constrained to sum to one, $\sum _{j=1}^{3}\epsilon _{j} = 1$), and *ϕ*(*x*;*μ*_*j*_,*σ*_*j*_) being the density of $\mathcal {N}(\mu _{j}, {\sigma _{j}^{2}})$. Furthermore, one component represents the empirical null distribution with its estimated mean (i.e., bias) and standard deviation (i.e., inflation). We propose to use a Gibbs sampling algorithm [31, 52, 53] to estimate the parameters of the mixture distribution.

Conjugate prior distributions are used for the means, *μ*_*j*_, variances, ${\sigma _{j}^{2}}$, and mixture proportions, *ε*_*j*_. Hence, we assume a normal distribution, , for the means, an inverse gamma distribution, ${\sigma ^{2}_{j}} \sim \mathcal {IG}(\alpha _{j}, \beta _{j})$, for the variances, and a Dirichlet distribution, $(\epsilon _{1}, \epsilon _{2}, \epsilon _{3}) \sim \mathcal {D}(\gamma _{1}, \gamma _{2}, \gamma _{3})$, for the mixture proportions. Well chosen hyper-priors ensure that the occurrence of labeling switching is minimized; i.e., during sampling from the posterior, the null component is switched with one of the alternative components. That is, we take informative hyper-priors for means, the null component, *λ*_1_=0, and for the alternative components *λ*_2_=−3 and *λ*_3_=3 all *τ*’s are equal to 100. The hyper-priors for the variance parameters are equal for all components *α*=1.28 and *β*=0.36 and were taken from Raftery [54]. For the Dirichlet distribution, widely used uniform noninformative prior parameters were chosen: *γ*_1_=*γ*_2_=*γ*_3_=1. Furthermore, data-dependent starting values are used to start the algorithm at a good initial point. These are based on the median and median absolute deviation (MAD) of the test statistics. A burn-in period of 3000 iterations was used as well as 2000 subsequent samples to estimate the parameters of the mixture distribution using the mean.

Given test statistics *x*_*i*_ (z-scores or transformed to z-scores) for *i*=1,⋯,*p*, prior distributions with hyper-parameters, and starting values for the posterior distributions, the Gibbs sampling algorithm is run in the following way:

Iterate for *t*=1,⋯,5000,

1. Generate the missing (unobserved) data: $z_{ij} \sim \mathcal {M}(\tilde {p}_{ij})$ from a multinomial distribution, with parameter *p*_*ij*_=*ε*_*j*_ *ϕ*(*x*_*i*_;*μ*_*j*_,*σ*_*j*_), $\tilde {p}_{ij}$ represents the normalized proportion $\left (\sum _{j=1}^{3} = \tilde {p}_{ij} = 1\right)$.
2. Obtain  and
3. Generate samples from the posteriors according to:
  3 $$ {}\begin{aligned} & \epsilon_{j} \sim \mathcal{D}(\gamma_{j}+n_{j}),\\ &\mu_{j}|{\sigma_{j}^{2}} \sim \mathcal{N}\left(\frac{\lambda_{j}\tau_{j} + s_{j}}{n_{j} + \tau_{j}}, \frac{{\sigma_{j}^{2}}+s_{j}}{n_{j} + \tau_{j}}\right),\\ &\sigma^{-2}_{j} \sim \Gamma \left(\alpha\,+\,\frac{1}{2}(n_{j}+1), \left(\beta + \frac{1}{2}\tau_{j}(\mu_{j}-\lambda_{j})^{2} \,+\, \frac{1}{2}{s_{j}^{2}}\right)^{-1}\right). \end{aligned}  $$

The latter mimics sampling from an inverse gamma distribution. For clarity, an iteration superscript is omitted. We assume that 3000 iterations (burn-in period) are sufficient for the Markov properties to hold and that the samples from the conditional distributions can be assumed to be samples from the joint parameter distribution. We implemented the Gibbs sampling algorithm in *C* and can either use weighted multinomial sampling method for binned test statistics or a fast sampling method [55] if all individual test statistics are used (user-defined). Optionally, test statistics following a distribution different from the normal distribution can be used by transforming them to z-scores. For example, test statistics *w*_1_,⋯,*w*_*p*_ that follow under the null hypothesis a *χ* ^2^-distribution with *ν* degrees of freedom can be transformed to z-scores using $\Phi ^{-1}(F_{\chi ^{2}_{\nu }}(w_{i}))$ [16] (Additional file 3).

## Additional files

Additional file 1 Additional figures and tables. Additional Figures S1, S2, S3, S4, and S5. Additional **Tables S1.** and S2. (534 KB PDF)

Additional file 2 Additional text. In the Additional text we prove that genomic control is equivalent to the use of an empirical null distribution. Furthermore, a sketch of a proof is given to show that the omission of a variable introduces bias, and we briefly describe how we impute white blood cell counts. The last section describes all participants and institutes that were involved in the generation of the BIOS data collection. (202 KB PDF)

Additional file 3 Additional simulations. R code and documentation of all simulations described in the manuscript and some additional simulations. (923 KB HTML)

Additional file 4 Additional Excel **Table S3a.** Additional Excel Table S3a with output of significant results of the EWAS meta-analyses of age: effect sizes, standard error, *P* values, test statistics of all cohorts, and the meta-analysis results. (1750 KB XLSX)

Additional file 5 Additional Excel **Table S3b.** Additional Excel Table S3b with output of significant results of the EWAS meta-analyses of smoking: effect sizes, standard error, *P* values, test statistics of all cohorts, and the meta-analysis results. (133 KB XLSX)

Additional file 6 Additional Excel **Table S3c.** Additional Excel Table S3c with output of significant results of the TWAS meta-analyses of age: effect sizes, standard error, *P* values, test statistics of all cohorts, and the meta-analysis results. (121 KB XLSX)

Additional file 7 Additional Excel **Table S3d.** Additional Excel Table S3d with output of significant results of the TWAS meta-analyses of smoking: effect sizes, standard error, *P* values, test statistics of all cohorts, and the meta-analysis results. (40 KB PDF)
