# Supplementary material for: Controlling bias and inflation in epigenome- and transcriptome-wide association studies using the empirical null distribution
Source: Genome Biol. 2017 Jan 27;18:19. doi: 10.1186/s13059-016-1131-9 (PMC5273857; doi:10.1186/s13059-016-1131-9)
Supplement: Additional file 1 — Additional figures and tables. Additional Figures S1, S2, S3, S4, and S5. Additional Tables S1. and S2. (534 KB PDF) [file 13059_2016_1131_MOESM1_ESM.pdf]

ADDITIONAL FIGURES AND TABLES *"CONTROLLING BIAS AND INFLATION IN EPIGENOME- AND TRANSCRIPTOME-WIDE ASSOCIATION STUDIES USING THE EMPIRICAL NULL DISTRIBUTION"*

1. Additional Figures

Figure 1. **Bias in EWAS and TWAS** Histograms of test-statistics TWASs (left panels) and EWASs (right panels) performed on two cohorts LifeLines (LL) and Leiden Longevity Study (LLS) for the phenotypes age and smoking status. In each panel a standard normal distribution is plotted (green) and an empirical null distribution (brown) estimated using our Bayesian method.

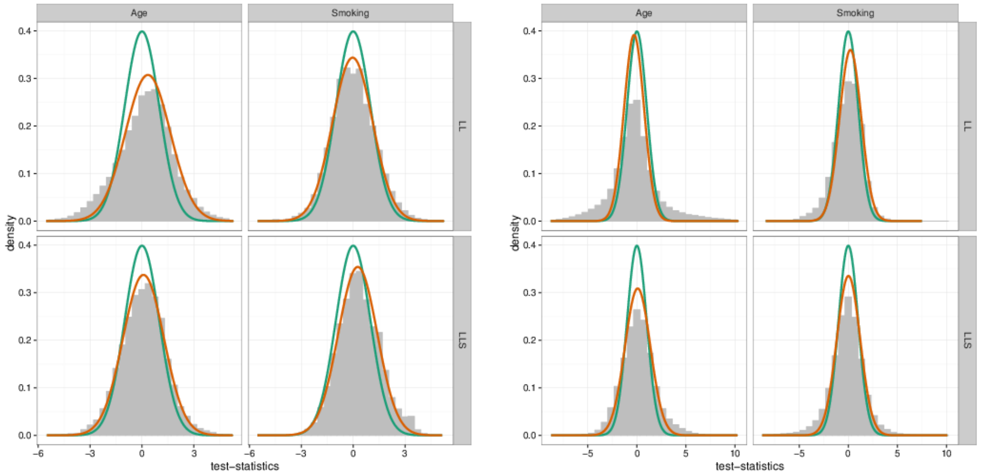

Date: January 4, 2017.

Figure 2. **Diagnostic plots for the Gibbs Sampler** This panel show diagnostic plots for the Gibbs Sampler run on the simulation scenario "equal". All plots are implemented in the BACON R package. A) Shows a trace-plot from the Gibbs Sampler for the estimated parameters of the three-component mixture model, with p the proportions, mu the means and sigma the standard deviations indexed with 0, 1 and 2 components (the 0 component represent the null distribution). All 5000 posterior estimates are shown although for parameter estimation a burnin period of 3000 is used. B) Fit for the estimated three component normal mixture to the 2000 samples from simulation scenario "equal". C) Scatter plot with normal confidence ellipses for the two, parameters proportion of null (p.0) features and inflation factor (sigma.0). Ellipses represent from the 70, 95 and 98 percent confidence intervals. D) Scatter plot with normal confidence ellipses for the two, parameters proportion of null (p.0) features and bias (mu.0). For more details see vignette of the package `https://bioconductor.org/packages/bacon/`.

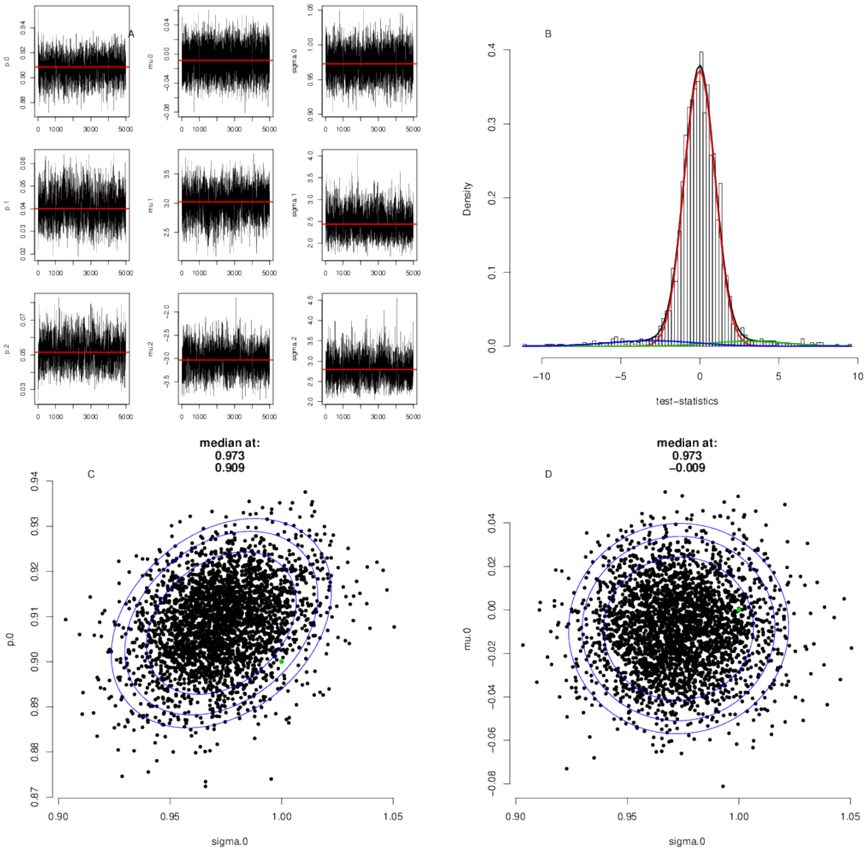

FIGURE 3. **Genomic inflation factor is affected by the proportion true associations** Box-plots summarizing the estimated inflation (y-axis) of 100 simulated sets of test-statistics with different amounts of true associations (x-axis). Inflation is estimated by five different methods: using our Bayesian method (dark green), the central moment matching method of Efron (brown) [1], $\sqrt{\lambda_{\chi_1^2}}$ (purple), the median absolute deviation (mad) of the test-statistics as proposed by Wang *et al.* [2] (dark-red) and the maximum likelihood approach of Efron (light green) [1]. The original genomic inflation factor ($\sqrt{\lambda_{\chi_1^2}}$) and the mad are severely biased by the amount of true associations present in the data, while the Bayesian inflation estimates are only mildly affected.

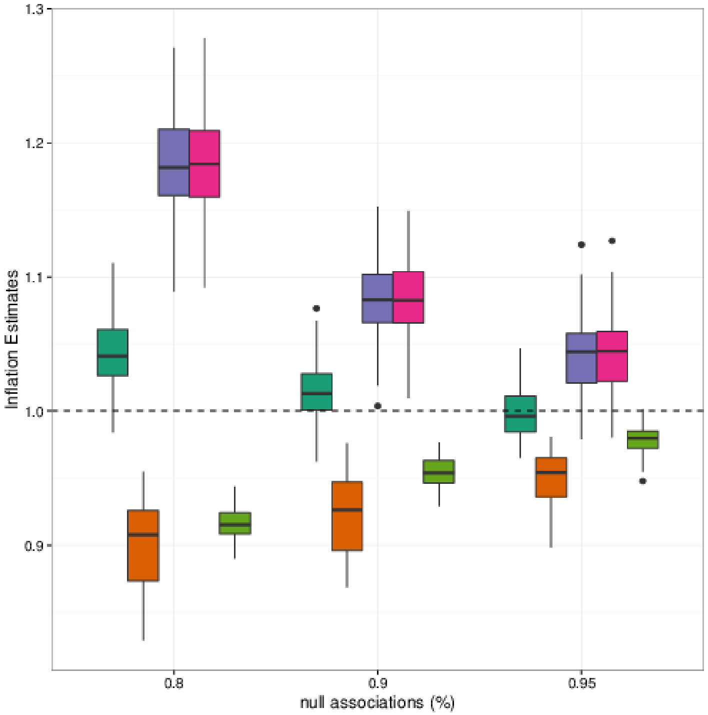

FIGURE 4. **Box-plots comparing the results of different methods to estimate an empirical null distribution under different scenarios** Sets of test-statistics were generated under different scenarios; scenario equal with equal proportion of positive and negative associations (0.05, 0.05), scenario skewed with only positive associations (0.1), scenario small is similar to scenario equal with only 0.01 true association, scenario close where the distribution for the means had expected value of 1 (in stead of 3). For each scenario 2000 test-statistics where generated 100 times (Methods). Under different scenarios the Bayesian method inflation factor estimate is competitive or better than existing methods that estimate an empirical null distribution including, central moment matching (CM) [1], maximum likelihood(MLE) [1] and median absolute deviation (mad) as proposed by Wang *et al.* [2]. The estimated parameters shown are prop0 (the proportion of null features), mu0 (mean of the empirical null distribution) and sigma0 (standard deviation of the empirical null distribution).

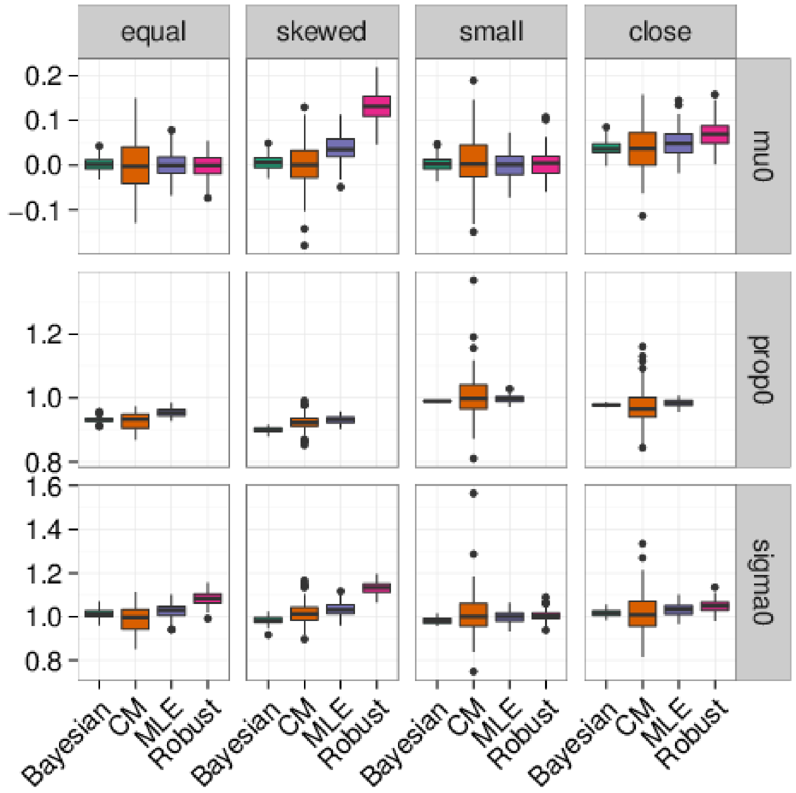

FIGURE 5. **Quantile-quantile plots for EWAS and TWAS meta-analyses across four cohorts of age and smoking status** Quantile-quantile plots summarize the results for the TWAS (panels A and B) and EWAS (C and D) meta-analysis across four cohorts; Cohort on Diabetes and Atherosclerosis Maastricht (CODAM), LifeLines (LL), Leiden Longevity Study (LLS), the Rotterdam Study (RS), on age (panels A and C) and smoking status (B and D). In each plot (A, B, C and D) left-panels represents bias and inflation uncorrected quantile quantile-plots while the right-panels the bias and inflation corrected results.

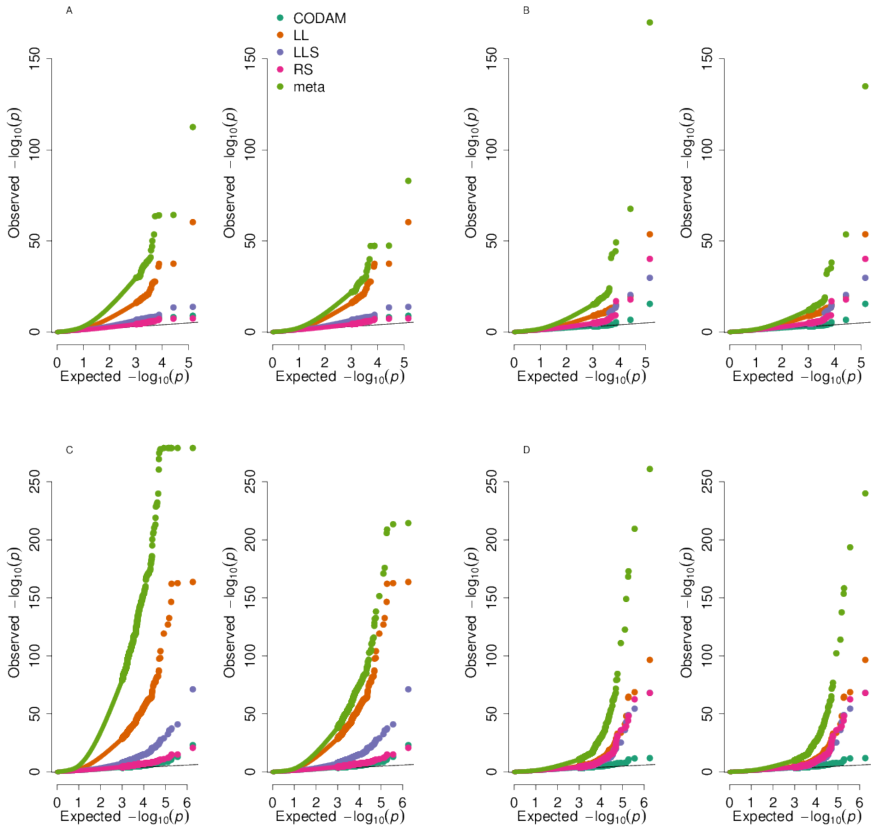

2. Additional Tables

Table 1. **Demographic variables cohorts** Distribution of age, sex and smoking status within the four cohorts; Cohort on Diabetes and Atherosclerosis Maastricht (CODAM, n≈180), LifeLines (LL, n≈700), Leiden Longevity Study (LLS, n≈600), the Rotterdam Study (RS, n≈600).

	CODAM	LL	LLS	RS
Median age:	66	46	59	69
interquartile range	61-71	35-55	55-64	67-72
Proportion female sex:	0.46	0.58	0.53	0.58
Smoking status (prop.):				
Never smoker	0.26	0.47	0.32	0.35
Former smoker	0.59	0.39	0.55	0.56
Current smoker	0.15	0.15	0.13	0.09

Table 2. **Correction for unobserved covariates reduces test-statistic bias and inflation** Genomic inflation factor estimates ($\sqrt{\lambda_{\chi_1^2}}$) and inflation factor (and bias) estimates using our Bayesian method from test-statistics obtained by fitting linear models for a EWAS on age in the Leiden Longevity Study (LLS) cohort subset of 500 individuals. Eight different models were fitting using different methods to estimate and correct for unobserved covariates: 1) only the covariate of interest, 2) including known covariates 3), 4), and 5) known covariates plus one, two and three principal component(s), 6) known covariates plus one optimal surrogate variables estimated using *iSVA* [3], 7) known covariates plus *RUVm* [4] and 8) known covariates plus plus 3 latent variables estimated using *CATE* [2] (within parentheses the number of principal components or optimal number of surrogate variables or optimal number of latent factors).

Method	Genomic inf. factor $\sqrt{\lambda_{\chi_1^2}}$	Bayesian infl. factor (bias)
1. No	1.692	1.536 (-0.011)
2. Known	1.524	1.320 (0.089)
3. PC(1)	1.390	1.245 (-0.239)
4. PC(2)	1.197	1.129 (0.049)
5. PC(3)	1.235	1.161 (0.051)
6. iSVA(3)	1.346	1.074 (0.057)
7. RUVm(-)	1.210	1.144 (-0.082)
8. CATE(3)	1.327	1.191 (0.131)

References

[1] Efron, B.: Large-scale simultaneous hypothesis testing: The choice of a null hypothesis. JASA **99**(465) (2004)

[2] Wang, J., Zhao, Q., Hastie, T., Owen, A.B.: Confounder Adjustment in Multiple Hypothesis Testing. ArXiv e-prints (2015)

[3] Teschendorff, A.E., Zhuang, J., Widschwendter, M.: Independent surrogate variable analysis to deconvolve confounding factors in large-scale microarray profiling studies. Bioinformatics **27**(11), 1496–1505 (2011)

[4] Maksimovic, J., Gagnon-Bartsch, J.A., Speed, T.P., Oshlack, A.: Removing unwanted variation in a differential methylation analysis of Illumina HumanMethylation450 array data. Nucleic Acids Res. **43**(16), 106 (2015)
