# Supplementary material for: Controlling bias and inflation in epigenome- and transcriptome-wide association studies using the empirical null distribution
Source: Genome Biol. 2017 Jan 27;18:19. doi: 10.1186/s13059-016-1131-9 (PMC5273857; doi:10.1186/s13059-016-1131-9)
Supplement: Additional file 2 — Additional text. In the Additional text we prove that genomic control is equivalent to the use of an empirical null distribution. Furthermore, a sketch of a proof is given to show that the omission of a variable introduces bias, and we briefly describe how we impute white blood cell counts. The last section describes all participants and institutes that were involved in the generation of the BIOS data collection. (202 KB PDF) [file 13059_2016_1131_MOESM2_ESM.pdf]

ADDITIONAL TEXT *"CONTROLLING BIAS AND INFLATION IN EPIGENOME- AND TRANSCRIPTOME-WIDE ASSOCIATION STUDIES USING THE EMPIRICAL NULL DISTRIBUTION"*

Abstract. Here we proof that genomic control is equivalent to the use of an empirical null distribution. Furthermore, a sketch of a proof is given to show that the omission of a variable introduces bias and we give a brief description how we impute white blood cell counts.

Contents

1. Genomic control is similar to an inflated or overdispersed null: 1
2. Unobserved covariates introduce bias and inflation 2
2.1. Omission of a variable introduces bias 2
3. Partial least squares for imputation of white blood cell composition 3
References 3

1. Genomic control is similar to an inflated or overdispersed null:

To test the hypothesis $H_{0i} : \beta_{1i} = 0$ we use the regular test statistic:

$$T_i = \frac{\hat{\beta}_{1i}}{\mathrm{SE}(\hat{\beta}_{1i})} \sim t_{n-2} \approx \mathcal{N}(0,1), \tag{1}$$

and assume that we can approximate the Student's t-distribution by the standard normal.

Genomic control divides the test-statistic by the inflation factor before the calculation of P values. The following derivation shows that applying genomic control is identical to the use of an overdispersed or inflated null. Consider, the interest is in two-sided P-values U_i then:

$$\begin{aligned} U_i &= 2\left[1 - \Phi\left(\left|\frac{T_i}{\lambda}\right|\right)\right] \\ &= 2\left[1 - \Pr\left\{Z \leq \left|\frac{T_i}{\lambda}\right|\right\}\right], \\ &= 2\left[1 - \Pr\left\{\lambda Z \leq |T_i|\right\}\right] \\ &= 2\left[1 - \Pr\left\{X \leq |T_i|\right\}\right] \end{aligned} \tag{2}$$

here, $Z \sim \mathcal{N}(0,1)$ with CDF given by $\Phi(\cdot)$. Furthermore, $X \sim \mathcal{N}(0, \lambda^2)$, represents the overdispersed or inflation normal distribution. Here λ is $\sqrt{\lambda_{\chi_1^2}}$ as defined in the main text.

Date: January 4, 2017.

And in general if both inflation and bias are present.

$$
\begin{aligned}
U_i &= 2\left[1 - \Phi\left(\left|\frac{T_i - \mu}{\sigma}\right|\right)\right] \\
&= 2\left[1 - \Pr\left\{\sigma Z + \mu \leq |T_i|\right\}\right]' \\
&= 2\left[1 - \Pr\left\{X \leq |T_i|\right\}\right]
\end{aligned} \tag{3}
$$

here, μ and σ represent the bias and inflation. Furthermore, now $X \sim \mathcal{N}(\mu, \sigma^2)$.

2. Unobserved covariates introduce bias and inflation

In a note from P. Rao [1] it was shown that the omission of a variable introduces bias and decreases the variance of all least squares estimates, i.e. introduces both bias and inflation.

2.1. **Omission of a variable introduces bias.** Here is a sketch of the proof for the introduction of bias, within the framework of the main text.

Considered the omission of the unobserved technical or biological covariates, $\mathbf{W}$. For the sake of simplicity, we assume there are no known covariates, $\mathbf{Z}$.

$$
\begin{aligned}
\mathbf{y}_j &= \mathbf{x}\tilde{\beta}_j + \tilde{\boldsymbol{\epsilon}}_{.j} \\
\mathbf{y}_j &= \mathbf{x}\beta_j + \mathbf{W}\boldsymbol{\gamma}_j + \boldsymbol{\epsilon}_{.j}
\end{aligned} \tag{4}
$$

The latter model is true but we are unaware of this and continue estimating the regression coefficient of interest, $\tilde{\beta}_j$ of the former, misspecified, model.

$$
\begin{aligned}
\tilde{b}_j &= \frac{\mathbf{x}^T\mathbf{y}_j}{\mathbf{x}^T\mathbf{x}} \\
&= \frac{\mathbf{x}^T(\mathbf{x}\beta_j + \mathbf{W}\boldsymbol{\gamma}_j + \boldsymbol{\epsilon}_{.j})}{\mathbf{x}^T\mathbf{x}} \\
&= \beta_j + \frac{\mathbf{x}^T\mathbf{W}\boldsymbol{\gamma}_j + \mathbf{x}^T\boldsymbol{\epsilon}_{.j}}{\mathbf{x}^T\mathbf{x}}
\end{aligned} \tag{5}
$$

where, we substituted the true model for $\mathbf{y}_j$. Now, since $E[\boldsymbol{\epsilon}_{.j}] = 0$, the expected regression coefficient is given by:

$$
E[\tilde{b}_j] = \beta_j + \frac{\mathbf{x}^T\mathbf{W}}{\mathbf{x}^T\mathbf{x}}\boldsymbol{\gamma}_j \tag{6}
$$

and the bias is given by the last fraction, which can be interpreted as a weighted sum of correlations between the covariate of interest, $\mathbf{x}$ and q omitted variables.

$$
\sum_k^q \gamma_{jk}\text{cor}(\mathbf{x}, \mathbf{w}_k) \tag{7}
$$

If all weights are zero or all correlations are zero than the bias will be zero too. The bias is not equal to zero if an omitted variable is confounded with the outcome, i.e., both γ_{jk} and $\mathbf{x}^T\mathbf{W}$ are not zero.

3. Partial least squares for imputation of white blood cell composition

White blood cell counts (WBC), i.e., neutrophils, lymphocytes, monocytes, eosinophils and basophils, were measures by the standard WBC differential as part of the CBC (Complete Blood Count). However, a minority of samples lack CBC measurements. Since DNA methylation levels are informative of the white blood cell composition [2] we build a linear predictor to infer the white blood cell composition of those samples lacking WBC measurements.

Obviously, this model can not be fitted using ordinary least-squares, since $p >> n$, we need some kind of regularization. Furthermore, the multivariate responses, white blood counts on five cell types, represents compositional data, i.e., the data are percentages that sum up to 100%.

We have chosen to use partial least-squares for fitting a model with cell counts as a multivariate response and the > 400.000 CpGs age and sex as covariates. It is known that the WBCC is dependent on age and gender. The advantage of partial least-squares is that it both can handle multivariate responses and high-dimensional ($p >> n$) covariates. We used the R-package pls [3] to fit the model and optimize the number of pls-components using five-fold cross-validation. The fitted model was used to predict WBCC using the 450K data, age and sex of those samples lacking WBCC.

The pls-approach has been validated by splitting the data with WBCC available in a train and test set. Fit the pls-model on the train set and predict WBCC on the test set. Correlation (Pearson) between predicted and measured WBCC range from $0.86 - 0.37$ for lymphocytes and basophils respectively, the intraclass correction was $0.84 - 0.25$.

This approach has been implemented in a R package wbccPredictor.

References

[1] Rao, P.: Some notes on misspecification in multiple regression. The American Statistican **25**(5) (1971)

[2] Houseman, E.A., *et al.*: DNA methylation arrays as surrogate measures of cell mixture distribution. BMC Bioinformatics **13**, 86 (2012)

[3] Mevik, B.H., Wehrens, R.: The pls Package: Principal Component and Partial Least Squares Regression in R. Journal of Statistical Software **18**(2) (2007)
