# Supplementary material for: Controlling bias and inflation in epigenome- and transcriptome-wide association studies using the empirical null distribution
Source: Genome Biol. 2017 Jan 27;18:19. doi: 10.1186/s13059-016-1131-9 (PMC5273857; doi:10.1186/s13059-016-1131-9)
Supplement: Additional file 3 — Additional simulations. R code and documentation of all simulations described in the manuscript and some additional simulations. (923 KB HTML) [file 13059_2016_1131_MOESM3_ESM.html]

Extended Simulations: Controlling bias and inflation in epigenome- and transcriptome-wide association studies using the empirical null distribution


# Extended Simulations: *Controlling bias and inflation in epigenome- and transcriptome-wide association studies using the empirical null distribution*

#### *Maarten van Iterson, Erik van Zwet and Bastiaan Heijmans*

#### *21 November 2016*

#### Abstract

This document describes several simulation studies to show the performance and robustness of BACON under various scenario’s and in comparison with other approaches that estimate the empirical null distribution or the amount of inflation.

#### Package version: bacon 1.2.0

# Contents

- 1 The genomic inflation factor overestimates inflation
  - 1.1 Compare with other methods
- 2 Estimation of the empirical null using different methods in various scenario’s
- 3 Extending simulations
  - 3.1 Simulate with bias
  - 3.2 Simulate with inflation
  - 3.3 Simulate with block-correlation structure among features
  - 3.4 Simulate from three-component normal mixture
  - 3.5 Simulation example from `Computer Age Statistical Inference`
  - 3.6 Simulate with large proportion of non-nulls
- 4 Applying `BACON` to non-normal data
  - 4.1 `edgeR` quasi-likelihood example
  - 4.2 Simulated non-normal data
- 5 Using simulated data cate
- 6 The effect of different prior parameters
- 7 Acknowledgement
- References

Global settings for the number of simulations and features used in all examples. In the paper the number of simulations was set to 100 and the number of features at 2000. Here we have extended this. Furthermore, since BACON version 1.1.7 the priors have been adjusted to cover a wider range of simulation scenario’s.

```
nsim <- 100
p <- 2000
priors = list(sigma = list(alpha = 1.28, beta = 0.36), mu = list(lambda = c(0, 
    3, -3), tau = c(100, 100, 100)), epsilon = list(gamma = c(1, 1, 1)))
```

# 1 The genomic inflation factor overestimates inflation

This simulation shows that the genomic inflation factor(Devlin and Roeder 1999), \(\lambda\_{\chi^2\_1}\), overestimates the amount of inflation when a moderated proportion of true associations is present.

```
f <- function(x) {
    sqrt(qchisq(1/(2 * x), 1)/qchisq(1/2, 1))
}
set.seed(12345)
theta1 <- c(0.8, 0, 1, 0, 3, 1)
theta2 <- c(0.9, 0, 1, 0, 3, 1)
theta3 <- c(0.95, 0, 1, 0, 3, 1)
sim.data1 <- replicate(nsim, rnormmix(p, theta1))  ##simulate
sim.data2 <- replicate(nsim, rnormmix(p, theta2))
sim.data3 <- replicate(nsim, rnormmix(p, theta3))
gif1 <- apply(sim.data1, 2, function(x) sqrt(median(x^2)/qchisq(1/2, 1)))  ##estimate
gif2 <- apply(sim.data2, 2, function(x) sqrt(median(x^2)/qchisq(1/2, 1)))
gif3 <- apply(sim.data3, 2, function(x) sqrt(median(x^2)/qchisq(1/2, 1)))
data <- data.frame(inflation = c(gif1, gif2, gif3), proportion = rep(c(0.8, 
    0.9, 0.95), each = nsim), group = factor(rep(c(0.8, 0.9, 0.95), each = nsim)))
gp <- ggplot(data, aes(x = proportion, y = inflation, group = group))  ##plot
gp <- gp + geom_boxplot()
gp <- gp + geom_hline(yintercept = 1, linetype = 2)
gp <- gp + stat_function(fun = f, colour = "red")
gp <- gp + ylab("Genomic Inflation Factor Estimates")
gp <- gp + theme_set(theme_bw(base_size = 20, base_family = "Helvetica")) + 
    theme_update(plot.margin = unit(rep(2, 4), units = "points"))
gp
```

This figure should be identical to Fig. 2 of the manuscript. The genomic inflation factor overestimates the amount of inflation even for moderated amount of true associations and in the absence of true inflation.

Back to top

## 1.1 Compare with other methods

Other methods have been proposed for the estimation of the empirical null distribution e.g., (B Efron 2004) Estimates for the mean and standard deviation of the empirical null distribution are estimates of bias and inflation. Furthermore, robust estimators are proposed for the estimation of bias and inflation e.g., using the median and mad (median absolute deviation)(Wang et al. 2015).

```
set.seed(12345)
theta1 <- c(0.8, 0, 1, 0, 3, 1)
theta2 <- c(0.9, 0, 1, 0, 3, 1)
theta3 <- c(0.95, 0, 1, 0, 3, 1)
sim.data1 <- replicate(nsim, rnormmix(p, theta1))  ##simulate
sim.data2 <- replicate(nsim, rnormmix(p, theta2))
sim.data3 <- replicate(nsim, rnormmix(p, theta3))
gif1 <- sqrt(apply(sim.data1^2, 2, median)/0.456)  ##genomic inflation factor Devlin and Roeder
gif2 <- sqrt(apply(sim.data2^2, 2, median)/0.456)
gif3 <- sqrt(apply(sim.data3^2, 2, median)/0.456)
mad1 <- apply(sim.data1, 2, mad)  ##mad propose by Wang et al.
mad2 <- apply(sim.data2, 2, mad)
mad3 <- apply(sim.data3, 2, mad)
mlecm1 <- apply(sim.data1, 2, function(x) locfdr(x, plot = 0)$fp0[c(3, 5), 3])  ##locfdr Efron et al.
mlecm2 <- apply(sim.data2, 2, function(x) locfdr(x, plot = 0)$fp0[c(3, 5), 3])
mlecm3 <- apply(sim.data3, 2, function(x) locfdr(x, plot = 0)$fp0[c(3, 5), 3])
bac1 <- apply(sim.data1, 2, function(x) inflation(bacon(x)))  ##bacon
bac2 <- apply(sim.data2, 2, function(x) inflation(bacon(x)))
bac3 <- apply(sim.data3, 2, function(x) inflation(bacon(x)))
data <- data.frame(inflation = c(gif1, gif2, gif3, mad1, mad2, mad3, mlecm1[1, 
    ], mlecm2[1, ], mlecm3[1, ], mlecm1[2, ], mlecm2[2, ], mlecm3[2, ], bac1, 
    bac2, bac3), proportion = factor(rep(rep(c(0.8, 0.9, 0.95), each = nsim), 
    5)), method = factor(rep(c("gif", "mad", "mle", "cm", "bif"), each = 3 * 
    nsim)))
gp <- ggplot(data, aes(x = proportion, y = inflation, fill = method))  ##create boxplots
gp <- gp + geom_boxplot()
gp <- gp + geom_hline(yintercept = 1, linetype = 2)
gp <- gp + xlab("null associations (%)") + ylab("Inflation Estimates")
gp <- gp + theme_set(theme_bw(base_size = 20, base_family = "Helvetica")) + 
    scale_fill_brewer(palette = "Dark2") + scale_color_brewer(palette = "Dark2")
gp
```

Here bif and gif represent the Bayesian inflation factor as estimated by *bacon* and the genomic inflation factor as proposed by (Devlin and Roeder 1999).

The figure should be identical to Additional Fig. 4 of the manuscript.

Back to top

# 2 Estimation of the empirical null using different methods in various scenario’s

Here we compare estimation of the empirical null distribution under different scenario’s using different approach:

1. Bayesian approach implemented in *bacon*
2. Robust estimation using the `median` and `mad` as proposed by (Wang et al. 2015)
3. Maximum likelihood approach of Efron implemented in *locfdr*
4. Central moment matching approach of Efron implemented in *locfdr*

```
simulate <- function(nsim, p, theta, name) {
    require(bacon)
    require(locfdr)
    require(matrixStats)
    sim.data <- replicate(nsim, rnormmix(p, theta))
    bc <- bacon(sim.data)
    bay <- estimates(bc)[, c(1, 4, 7)]
    rob <- cbind(NA, colMedians(sim.data), colMads(sim.data))
    mle <- t(apply(sim.data, 2, function(x) locfdr(x, plot = 0)$fp0[3, c(3, 
        1, 2)]))
    cm <- t(apply(sim.data, 2, function(x) locfdr(x, plot = 0)$fp0[5, c(3, 1, 
        2)]))
    data.frame(values = c(as.vector(bay), as.vector(mle), as.vector(cm), as.vector(rob)), 
        estimate = rep(rep(c("prop0", "mu0", "sigma0"), each = nsim), 4), method = rep(c("Bayesian", 
            "MLE", "CM", "Robust"), each = 3 * nsim), scenario = rep(name, 3 * 
            4 * nsim))
}
set.seed(12345)
thetas <- list(equal = c(0.9, 0, 1, 0, 3, 1), skewed = c(0.9, 0, 1, 3, 1, 1), 
    small = c(0.98, 0, 1, 0, 1, 1), close = c(0.9, 0, 1, 1, 1, 1))
data <- c()
for (name in names(thetas)) data <- rbind(data, simulate(nsim, p, thetas[[name]], 
    name))
```

```
## Detected 2 workers!
##  Running in parallel!
## Detected 2 workers!
##  Running in parallel!
## Detected 2 workers!
##  Running in parallel!
## Detected 2 workers!
##  Running in parallel!
```

```
gp <- ggplot(data, aes(x = method, y = values, fill = method))  ##plotting
gp <- gp + geom_boxplot()
gp <- gp + facet_grid(estimate ~ scenario, scales = "free_y")
gp <- gp + scale_fill_brewer(palette = "Dark2") + scale_color_brewer(palette = "Dark2")
gp <- gp + xlab(label = "") + ylab(label = "")
gp <- gp + theme_set(theme_bw(base_size = 20, base_family = "Helvetica")) + 
    theme_update(plot.margin = unit(rep(2, 4), units = "points"))
gp <- gp + theme(legend.position = "none") + theme(axis.text.x = element_text(angle = 45, 
    hjust = 1))
gp
```

This figure is identical to Additional Fig. 5 of the manuscript.

Back to top

# 3 Extending simulations

## 3.1 Simulate with bias

Here we simulate test-statistic with a strong positive or negative bias to investigate how flexible the Bayesian estimation of the empirical null is.

```
set.seed(12345)
thetapos <- c(0.9, 1, 1, 0, 3, 1)
thetaneg <- c(0.9, -1, 1, 0, 3, 1)
estpos <- estneg <- matrix(nrow = nsim, ncol = 9)
for (i in 1:nsim) {
    z <- rnormmix(p, thetapos)
    estpos[i, ] <- estimates(bacon(z, priors = priors))
    z <- rnormmix(p, thetaneg)
    estneg[i, ] <- estimates(bacon(z, priors = priors))
}
results <- rbind(c(thetapos[1], NA, NA, thetapos[2], NA, NA, thetapos[3], NA, 
    NA), apply(estpos, 2, mean), apply(estpos, 2, sd), apply(estneg, 2, mean), 
    apply(estneg, 2, sd))
colnames(results) <- colnames(estimates(bacon(z, priors = priors)))
rownames(results) <- c("true", "mean (pos.)", "stdev (pos.)", "mean (neg.)", 
    "stdev (neg.)")
results <- signif(results, 3)
results[1, 4] <- paste0("+/-", results[1, 4])
kable(results)
```

|  | p.0 | p.1 | p.2 | mu.0 | mu.1 | mu.2 | sigma.0 | sigma.1 | sigma.2 |
| --- | --- | --- | --- | --- | --- | --- | --- | --- | --- |
| true | 0.9 | NA | NA | +/-1 | NA | NA | 1 | NA | NA |
| mean (pos.) | 0.934 | 0.0334 | 0.0325 | 0.913 | 2.99 | -3.03 | 1.05 | 1.83 | 1.77 |
| stdev (pos.) | 0.00994 | 0.00826 | 0.00574 | 0.0264 | 0.0175 | 0.0369 | 0.0231 | 0.329 | 0.296 |
| mean (neg.) | 0.933 | 0.0329 | 0.0339 | -0.911 | 3.02 | -2.99 | 1.05 | 1.82 | 1.83 |
| stdev (neg.) | 0.00985 | 0.00474 | 0.00774 | 0.0276 | 0.0337 | 0.0129 | 0.0201 | 0.269 | 0.319 |

```
fit(bacon(z, priors = priors), n = 25, main = "neg. bias fit (colored) and true (black)", 
    xlim = c(-6, 6))
curve(0.9 * dnorm(x, -1, 1), add = TRUE, col = 1, lwd = 2, lty = 2)
```

Although the null is not exactly identical it is very close even with the strong bias.

Back to top

## 3.2 Simulate with inflation

Here we simulate test-statistic with a strong positive or negative bias to investigate how flexible the Bayesian estimation of the empirical null is.

```
set.seed(12345)
thetainfl <- c(0.9, 0, 1.2, 0, 3, 1)
thetadefl <- c(0.9, 0, 0.8, 0, 3, 1)
estinfl <- estdefl <- matrix(nrow = nsim, ncol = 9)
for (i in 1:nsim) {
    z <- rnormmix(p, thetainfl)
    estinfl[i, ] <- estimates(bacon(z, priors = priors))
    z <- rnormmix(p, thetadefl)
    estdefl[i, ] <- estimates(bacon(z, priors = priors))
}
results <- rbind(c(thetainfl[1], NA, NA, thetainfl[2], NA, NA, thetainfl[3], 
    NA, NA), apply(estinfl, 2, mean), apply(estinfl, 2, sd), apply(estdefl, 
    2, mean), apply(estdefl, 2, sd))
colnames(results) <- colnames(estimates(bacon(z, priors = priors)))
rownames(results) <- c("true", "mean (infl.)", "stdev (infl.)", "mean (defl.)", 
    "stdev (defl.)")
results <- signif(results, 3)
results[1, 7] <- paste(thetainfl[3], thetadefl[3], sep = "/")
kable(results)
```

|  | p.0 | p.1 | p.2 | mu.0 | mu.1 | mu.2 | sigma.0 | sigma.1 | sigma.2 |
| --- | --- | --- | --- | --- | --- | --- | --- | --- | --- |
| true | 0.9 | NA | NA | 0 | NA | NA | 1.2/0.8 | NA | NA |
| mean (infl.) | 0.933 | 0.0328 | 0.0337 | -0.000831 | 2.99 | -3 | 1.2 | 1.84 | 1.83 |
| stdev (infl.) | 0.00973 | 0.00729 | 0.00644 | 0.0303 | 0.0161 | 0.0172 | 0.0264 | 0.306 | 0.316 |
| mean (defl.) | 0.931 | 0.0348 | 0.0345 | 0.00257 | 2.98 | -2.98 | 0.82 | 1.85 | 1.8 |
| stdev (defl.) | 0.00799 | 0.00528 | 0.00585 | 0.0207 | 0.0316 | 0.0366 | 0.0157 | 0.264 | 0.249 |

```
fit(bacon(z, priors = priors), n = 25, main = "defl. fit (colored) and true (black)", 
    xlim = c(-6, 6))
curve(0.9 * dnorm(x, 0, 0.8), add = TRUE, col = 1, lwd = 2, lty = 2)
```

Although the null is not exactly identical it is very close even with the moderated inflation or deflation.

Back to top

## 3.3 Simulate with block-correlation structure among features

Here we use the approach of Efron to introduce correlation among genes(B. Efron 2010).

The following function generates two sets of test-statistics one with block-correlation structure one without correlation. Both sets have added for a proportion of the genes an effect according to our mixture simulation.

```
load(url("http://statweb.stanford.edu/~ckirby/brad/LSI/datasets-and-programs/programs/simz.RData"))
gendat <- function(N, theta, alpha) {
    U <- runif(N, min = 0, max = 1)
    N1 <- sum(U < theta[1])
    N2 <- sum(U >= theta[1])
    index <- rep(FALSE, N)
    index[sample(1:N, N2)] <- TRUE
    mu <- rep(0, N)
    mu[index] <- rnorm(N2, mean = theta[4], sd = theta[5])
    
    ## uncorrelated matrix genes
    z <- simz(alpha = 0, N, 1, stand = 1)
    zu <- mu + z
    ## correlated matrix genes
    z <- simz(alpha = alpha, N, 1, stand = 1)
    zc <- mu + z
    return(list(zu = zu, zc = zc, index = index))
}
```

Here we generate 100 times two sets of test-statistics one with a correlation structure among genes and one without.

```
set.seed(12345)
theta <- c(0.98, 0, 1, 3, 1, 1)
alpha <- 0.4
cor <- uncor <- matrix(nrow = nsim, ncol = 9)
for (i in 1:nsim) {
    ce <- gendat(p, theta, alpha)
    uncor[i, ] <- estimates(bacon(ce$zu))
    cor[i, ] <- estimates(bacon(ce$zc))
}
names <- rep(colnames(estimates(bacon(ce$zu))), each = nsim)
data <- data.frame(values = as.vector(uncor), estimates = gsub("\\..*$", "", 
    names), component = gsub("^.*\\.", "", names), group = "uncorrelated")
data <- rbind(data, data.frame(values = as.vector(cor), estimates = gsub("\\..*$", 
    "", names), component = gsub("^.*\\.", "", names), group = "correlated"))
```

For example, plot the estimate proportion of null genes, the mean and standard deviation of the null distribution for both the correlated and uncorrelated test-statistics.

```
gp <- ggplot(subset(data, component == 0), aes(component, values, colour = group))
gp <- gp + geom_boxplot() + geom_point(position = position_jitterdodge())
gp <- gp + facet_wrap(~estimates, scales = "free_y")
gp
```

Or tabulate the results.

```
results <- rbind(c(theta[1], NA, NA, theta[2], NA, NA, theta[3], NA, NA), apply(uncor, 
    2, mean), apply(uncor, 2, sd), apply(cor, 2, mean), apply(cor, 2, sd))
colnames(results) <- colnames(estimates(bacon(ce$zu)))
rownames(results) <- c("true", "mean (uncor)", "stdev (uncor)", "mean (cor)", 
    "stdev (cor)")
kable(round(results, 3))
```

|  | p.0 | p.1 | p.2 | mu.0 | mu.1 | mu.2 | sigma.0 | sigma.1 | sigma.2 |
| --- | --- | --- | --- | --- | --- | --- | --- | --- | --- |
| true | 0.900 | NA | NA | 0.000 | NA | NA | 1.000 | NA | NA |
| mean (uncor) | 0.926 | 0.051 | 0.022 | 0.002 | 3.012 | -2.972 | 1.009 | 2.167 | 1.514 |
| stdev (uncor) | 0.008 | 0.006 | 0.004 | 0.008 | 0.036 | 0.017 | 0.022 | 0.261 | 0.312 |
| mean (cor) | 0.928 | 0.050 | 0.022 | 0.002 | 3.023 | -2.958 | 0.957 | 2.145 | 1.485 |
| stdev (cor) | 0.014 | 0.014 | 0.011 | 0.049 | 0.072 | 0.121 | 0.141 | 0.340 | 0.497 |

Even with moderated correlation the estimates variance increases almost 10x for the mean and standard deviation. Although on average the estimated mean and proportion nulls is in good agreement with the expected values there is a observed deflation for the estimated standard deviation.

```
fpr <- tpr <- matrix(nrow = nsim, ncol = 6)
for (i in 1:nsim) {
    ce <- gendat(p, theta, alpha)
    Pvals <- matrix(nrow = p, ncol = 6)
    bc <- bacon(ce$zu, priors = priors)
    Pvals[, 1] <- pval(bc, corrected = FALSE)
    Pvals[, 2] <- pval(bc)
    t <- tstat(bc, corrected = FALSE)
    Pvals[, 3] <- 2 * pnorm(-abs(t/sqrt(median(t^2)/qchisq(1/2, 1))))
    bc <- bacon(ce$zc, priors = priors)
    Pvals[, 4] <- pval(bc, corrected = FALSE)
    Pvals[, 5] <- pval(bc)
    t <- tstat(bc, corrected = FALSE)
    Pvals[, 6] <- 2 * pnorm(-abs(t/sqrt(median(t^2)/qchisq(1/2, 1))))
    fpr[i, ] <- apply(Pvals[!ce$index, ], 2, function(x) mean(x < 0.05))  ##Type I error
    tpr[i, ] <- apply(Pvals[ce$index, ], 2, function(x) mean(x < 0.05))  ##Power
}
colnames(fpr) <- colnames(tpr) <- c("uncor (naive)", "uncor (emp)", "uncor (N(0,1))", 
    "cor(naive)", "cor (emp)", "cor (N(0,1))")
results <- rbind(signif(apply(fpr, 2, mean), 2), signif(apply(fpr, 2, sd), 1), 
    signif(apply(tpr, 2, mean), 2), signif(apply(tpr, 2, sd), 1))
rownames(results) <- c("Type I error (mean)", "Type I error (stdev)", "Power (mean)", 
    "Power (stdev)")
kable(results)
```

|  | uncor (naive) | uncor (emp) | uncor (N(0,1)) | cor(naive) | cor (emp) | cor (N(0,1)) |
| --- | --- | --- | --- | --- | --- | --- |
| Type I error (mean) | 0.050 | 0.053 | 0.029 | 0.038 | 0.050 | 0.023 |
| Type I error (stdev) | 0.005 | 0.005 | 0.005 | 0.020 | 0.008 | 0.007 |
| Power (mean) | 0.770 | 0.770 | 0.720 | 0.770 | 0.800 | 0.740 |
| Power (stdev) | 0.030 | 0.030 | 0.030 | 0.040 | 0.070 | 0.090 |

More or less the same trend is observed as with estimation of the empirical null. For correlated data the standard deviations are much larger while the expected Type I error is similar as estimated from uncorrelated data.

Back to top

## 3.4 Simulate from three-component normal mixture

Here we simulated from a three component normal mixture, i.e, same model that is used for fitting the data.

```
set.seed(12345)
theta <- c(0.8, 0.1, 0.1, 0, -3, 4, 1, 3, 2)  ##extended parameters compared to rnormmix
z <- c(rnorm(p * theta[1], theta[4], theta[7]), rnorm(p * theta[2], theta[5], 
    theta[8]), rnorm(p * theta[3], theta[6], theta[9]))
h <- hist(z, n = 25, freq = FALSE)
curve(theta[1] * dnorm(x, theta[4], theta[7]), add = TRUE)
curve(theta[2] * dnorm(x, theta[5], theta[8]), add = TRUE)
curve(theta[3] * dnorm(x, theta[6], theta[9]), add = TRUE)
```

```
set.seed(12345)
est <- matrix(nrow = nsim, ncol = 9)
for (i in 1:nsim) {
    z <- c(rnorm(p * theta[1], theta[4], theta[7]), rnorm(p * theta[2], theta[5], 
        theta[8]), rnorm(p * theta[3], theta[6], theta[9]))
    est[i, ] <- estimates(bacon(z, priors = priors))
}
names <- rep(colnames(estimates(bacon(z, priors = priors))), each = nsim)
data <- data.frame(values = as.vector(est), estimates = gsub("\\..*$", "", names), 
    component = gsub("^.*\\.", "", names))
```

```
data <- rbind(theta[c(1, 3, 2, 4, 6, 5, 7, 9, 8)], apply(est, 2, mean), apply(est, 
    2, sd))
colnames(data) <- colnames(estimates(bacon(z, priors = priors)))
rownames(data) <- c("true", "mn", "sd")
kable(signif(data, 3))
```

|  | p.0 | p.1 | p.2 | mu.0 | mu.1 | mu.2 | sigma.0 | sigma.1 | sigma.2 |
| --- | --- | --- | --- | --- | --- | --- | --- | --- | --- |
| true | 0.80000 | 0.10000 | 0.10000 | 0.0000 | 4.0000 | -3.0000 | 1.0000 | 2.000 | 3.000 |
| mn | 0.77100 | 0.13100 | 0.09820 | -0.0123 | 3.1300 | -3.0500 | 0.9710 | 2.320 | 2.750 |
| sd | 0.00774 | 0.00572 | 0.00598 | 0.0253 | 0.0547 | 0.0525 | 0.0253 | 0.116 | 0.197 |

Estimated parameters are in good agreement with the expected values.

Back to top

## 3.5 Simulation example from `Computer Age Statistical Inference`

Section 15.5 (B Efron and Hastie 2016) discusses briefly estimation of the empirical null distribution and describes a small simulation studies to give some ideas on the biases and variabilities inherent in the *locfdr* estimation process.

Here I use the same simulation setup but added bacon as competitive estimator of the empirical null.

```
set.seed(12345)
nsim <- 250
p <- 5000
sim.data1 <- replicate(nsim, c(rnorm(0.95 * p, 0, 1), rnorm(0.05 * p, 3, 1)))  ##simulate
sim.data2 <- replicate(nsim, c(rnorm(0.95 * p, 0, 1), rnorm(0.05 * p, 4.2, 1)))
mlecm1 <- apply(sim.data1, 2, function(x) locfdr(x, plot = 0)$fp0[c(3, 5), ])  ##locfdr Efron et al.
mlecm2 <- apply(sim.data2[, 1:5], 2, function(x) locfdr(x, plot = 0)$fp0[c(3, 
    5), ])
bac1 <- apply(sim.data1, 2, function(x) estimates(bacon(x))[c(1, 4, 7)])  ##bacon
bac2 <- apply(sim.data2, 2, function(x) estimates(bacon(x))[c(1, 4, 7)])
results1 <- rbind(c(theta1[2], theta1[3], theta1[1]), matrix(apply(mlecm1, 1, 
    mean), 2, 3), apply(bac1, 1, mean)[c(2, 3, 1)], matrix(apply(mlecm1, 1, 
    sd), 2, 3), apply(bac1, 1, sd)[c(2, 3, 1)])
colnames(results1) <- c("delta0", "sigma0", "pi0")
rownames(results1) <- c("true", paste(rep(c("mean", "stdev"), each = 3), rep(c("(mle)", 
    "(ce)", "(bacon)"), 2)))
results2 <- rbind(c(theta2[2], theta2[3], theta2[1]), matrix(apply(mlecm2, 1, 
    mean), 2, 3), apply(bac2, 1, mean)[c(2, 3, 1)], matrix(apply(mlecm2, 1, 
    sd), 2, 3), apply(bac2, 1, sd)[c(2, 3, 1)])
colnames(results2) <- c("delta0", "sigma0", "pi0")
rownames(results2) <- c("true", paste(rep(c("mean", "stdev"), each = 3), rep(c("(mle)", 
    "(ce)", "(bacon)"), 2)))
results <- cbind(results1, results2)
round(results, 3)
```

```
##               delta0 sigma0   pi0 delta0 sigma0   pi0
## true           0.000  1.000 0.800  0.000  1.000 0.900
## mean (mle)     0.010  1.013 0.960  0.009  0.987 0.947
## mean (ce)      0.002  0.974 0.934  0.057  0.948 0.923
## mean (bacon)   0.006  0.990 0.950 -0.022  0.964 0.931
## stdev (mle)    0.019  0.022 0.010  0.018  0.015 0.008
## stdev (ce)     0.034  0.040 0.024  0.026  0.029 0.017
## stdev (bacon)  0.012  0.012 0.003  0.012  0.011 0.002
```

A few results are interesting *bacon* seems to have the smallest standard deviation, while the central moment matching approach the largest. The mean estimates are as close or closer to the expected values as the maximum likelihood approach.

We could add some other examples from Efron since all data sets are available???

Back to top

## 3.6 Simulate with large proportion of non-nulls

Run simulation with large, say, 40% proportion of non-nulls.

```
theta <- c(0.6, 0, 1, 1, 3, 1)
est <- matrix(nrow = nsim, ncol = 9)
for (i in 1:nsim) {
    z <- rnormmix(p, theta)
    est[i, ] <- estimates(bacon(z, priors = priors))
}
names <- rep(colnames(estimates(bacon(z, priors = priors))), each = nsim)
data <- data.frame(values = as.vector(est), estimates = gsub("\\..*$", "", names), 
    component = gsub("^.*\\.", "", names))
theta <- c(0.6, NA, NA, 0, NA, NA, 1, NA, NA)
data <- rbind(theta, apply(est, 2, mean), apply(est, 2, sd))
colnames(data) <- colnames(estimates(bacon(z, priors = priors)))
rownames(data) <- c("true", "mean", "stdev")
kable(signif(data, 3))
```

|  | p.0 | p.1 | p.2 | mu.0 | mu.1 | mu.2 | sigma.0 | sigma.1 | sigma.2 |
| --- | --- | --- | --- | --- | --- | --- | --- | --- | --- |
| true | 0.6000 | NA | NA | 0.00000 | NA | NA | 1.0000 | NA | NA |
| mean | 0.7020 | 0.2220 | 0.07600 | 0.00624 | 2.830 | -2.9600 | 1.0700 | 2.4400 | 1.850 |
| stdev | 0.0135 | 0.0127 | 0.00693 | 0.02440 | 0.106 | 0.0523 | 0.0258 | 0.0914 | 0.121 |

Of course we can always adjust the prior parameters e.g., here we assign more weight to the alternative Dirichlet prior parameters.

```
priors = list(sigma = list(alpha = 1.28, beta = 0.36), mu = list(lambda = c(0, 
    3, -3), tau = c(100, 100, 100)), epsilon = list(gamma = c(1, 25, 25)))
estimates(bacon(z, priors = priors))
```

```
##            p.0       p.1        p.2        mu.0     mu.1      mu.2
## [1,] 0.6545217 0.2483022 0.09717605 -0.03387671 2.636836 -2.480639
##        sigma.0  sigma.1  sigma.2
## [1,] 0.9984402 2.505345 2.112102
```

```
bc <- bacon(z, priors = priors)
fit(bc, n = 50)
curve(0.6 * dnorm(x, 0, 1), lty = 2, add = TRUE, lwd = 2)
```

Bacon seems to under estimate the proportion of non-nulls in this situation. As a cost of increase in stdev of the null. But this will also depend on effect-sizes.

```
theta <- c(0.6, 0, 1, 2, 3, 1)
est <- matrix(nrow = nsim, ncol = 9)
for (i in 1:nsim) {
    z <- rnormmix(p, theta)
    est[i, ] <- estimates(bacon(z, priors = priors))
}
names <- rep(colnames(estimates(bacon(z, priors = priors))), each = nsim)
data <- data.frame(values = as.vector(est), estimates = gsub("\\..*$", "", names), 
    component = gsub("^.*\\.", "", names))
theta <- c(0.6, NA, NA, 0, NA, NA, 1, NA, NA)
data <- rbind(theta, apply(est, 2, mean), apply(est, 2, sd))
colnames(data) <- colnames(estimates(bacon(z, priors = priors)))
rownames(data) <- c("true", "mean", "stdev")
kable(signif(data, 3))
```

|  | p.0 | p.1 | p.2 | mu.0 | mu.1 | mu.2 | sigma.0 | sigma.1 | sigma.2 |
| --- | --- | --- | --- | --- | --- | --- | --- | --- | --- |
| true | 0.600 | NA | NA | 0.0000 | NA | NA | 1.0000 | NA | NA |
| mean | 0.635 | 0.3090 | 0.05560 | 0.0176 | 2.9700 | -2.6200 | 1.0100 | 2.6600 | 1.710 |
| stdev | 0.012 | 0.0112 | 0.00492 | 0.0233 | 0.0796 | 0.0558 | 0.0202 | 0.0637 | 0.149 |

Now simulate with also large effect-sizes.

```
theta <- c(0.6, 0, 1, 2, 4, 1)
est <- matrix(nrow = nsim, ncol = 9)
for (i in 1:nsim) {
    z <- rnormmix(p, theta)
    est[i, ] <- estimates(bacon(z, priors = priors))
}
names <- rep(colnames(estimates(bacon(z, priors = priors))), each = nsim)
data <- data.frame(values = as.vector(est), estimates = gsub("\\..*$", "", names), 
    component = gsub("^.*\\.", "", names))
theta <- c(0.6, NA, NA, 0, NA, NA, 1, NA, NA)
data <- rbind(theta, apply(est, 2, mean), apply(est, 2, sd))
colnames(data) <- colnames(estimates(bacon(z, priors = priors)))
rownames(data) <- c("true", "mean", "stdev")
kable(signif(data, 3))
```

|  | p.0 | p.1 | p.2 | mu.0 | mu.1 | mu.2 | sigma.0 | sigma.1 | sigma.2 |
| --- | --- | --- | --- | --- | --- | --- | --- | --- | --- |
| true | 0.600 | NA | NA | 0.0000 | NA | NA | 1.0000 | NA | NA |
| mean | 0.604 | 0.3030 | 0.09310 | 0.0116 | 3.290 | -2.4200 | 1.0000 | 3.4800 | 2.740 |
| stdev | 0.012 | 0.0102 | 0.00608 | 0.0218 | 0.093 | 0.0789 | 0.0229 | 0.0857 | 0.158 |

The estimated mixture distribution propely fits the data in this scenario.

Back to top

# 4 Applying `BACON` to non-normal data

## 4.1 `edgeR` quasi-likelihood example

Only possible via a transformation to z-values, e.g., Efron’s z-transformation.

Here is an example using count data from the *edgeR* vignette section 4.3 `Profiles of Yoruba HapMap individuals`. Test-statistic used here a quasi-likelihood ratio-test that takes genewise variances in account (empirical Bayes approach) which results that under the null hypothesis the test-statistic follow a F-distribution.

```
data(pickrell1)
Counts <- exprs(pickrell1.eset)
Gender <- pickrell1.eset$gender
rm(pickrell1.eset)
data(annotEnsembl63)
annot <- annotEnsembl63[, c("Symbol", "Chr")]
rm(annotEnsembl63)
y <- DGEList(counts = Counts, genes = annot[rownames(Counts), ])
isexpr <- rowSums(cpm(y) > 1) >= 20
hasannot <- rowSums(is.na(y$genes)) == 0
y <- y[isexpr & hasannot, , keep.lib.sizes = FALSE]
y <- calcNormFactors(y)
design <- model.matrix(~Gender)
y <- estimateDisp(y, design, robust = TRUE)
fit <- glmQLFit(y, design, robust = TRUE)
qlf <- glmQLFTest(fit)
res <- as.data.frame(topTags(qlf, n = nrow(counts)))
head(res)
```

```
##                   Symbol Chr     logFC   logCPM         F       PValue
## ENSG00000229807     XIST   X -9.485109 7.248644 1213.2655 1.030708e-46
## ENSG00000099749 CYorf15A   Y  4.279499 1.756924  856.6674 1.186779e-41
## ENSG00000131002 CYorf15B   Y  5.625815 2.055374  587.3207 2.660854e-36
## ENSG00000157828   RPS4Y2   Y  3.175600 4.207146  585.0200 3.017466e-36
## ENSG00000233864   TTTY15   Y  4.839482 1.253822  537.7832 4.421561e-35
## ENSG00000198692   EIF1AY   Y  2.359992 3.246548  375.5065 3.041696e-30
##                          FDR
## ENSG00000229807 1.784156e-42
## ENSG00000099749 1.027157e-37
## ENSG00000131002 1.305809e-32
## ENSG00000157828 1.305809e-32
## ENSG00000233864 1.530744e-31
## ENSG00000198692 8.775292e-27
```

Now we transform the F-statistics to z-score via P values, i.e., \(z = \Phi^{-1}(1 - F\_{F\_{\nu, 1}}(f))\). (Actually, P values are calculated with genewise differences in the degrees of freedom as a result of the empirical Bayes analysis).

```
hist(res$PValue)
```

```
z <- qnorm(1 - res$PValue)
hist(z, n = 50)
```

```
z[!is.finite(z)] <- 16
estimates(bacon(z, priors = priors))
```

```
##            p.0         p.1          p.2      mu.0     mu.1      mu.2
## [1,] 0.9947817 0.005087697 0.0001306095 0.1496109 3.093815 -2.992524
##       sigma.0 sigma.1   sigma.2
## [1,] 1.034135 2.17404 0.7901363
```

Is there a difference with using the linear model, e.g., when using `voom`.

```
v <- voom(y, design)
fit <- lmFit(v, design)
z <- fit$coef[, 2]/fit$stdev.unscaled[, 2]/fit$sigma
hist(z, n = 100, xlim = c(-10, 10))
```

```
estimates(bacon(z, priors = priors))
```

```
##            p.0          p.1        p.2       mu.0     mu.1      mu.2
## [1,] 0.9958433 0.0002456772 0.00391101 0.02409905 2.993919 -3.028117
##       sigma.0   sigma.1  sigma.2
## [1,] 1.082282 0.6567649 1.788893
```

Estimates are quite close the linear model approach is sligtly inflated, however, it also has a smaller bias.

Back to top

## 4.2 Simulated non-normal data

Squaring the original simulated z-statistics from the mixture distribution?

```
theta <- c(0.9, 0, 1, 0, 3, 1)
est <- estchi <- matrix(nrow = nsim, ncol = 9)
for (i in 1:nsim) {
    z <- rnormmix(p, theta)
    est[i, ] <- estimates(bacon(z, priors = priors))
    pval <- 1 - pchisq(z^2, 1)  ##p-values
    z <- qnorm(1 - pval)  ##z-values
    z[!is.finite(z)] <- 16
    estchi[i, ] <- estimates(bacon(z, priors = priors))
}
results <- rbind(c(theta[1], NA, NA, theta[2], NA, NA, theta[3], NA, NA), apply(est, 
    2, mean), apply(est, 2, sd), apply(estchi, 2, mean), apply(estchi, 2, sd))
colnames(results) <- colnames(estimates(bacon(z, priors = priors)))
rownames(results) <- c("true", "mean (z)", "stdev (z)", "mean (z^2)", "stdev (z^2)")
kable(signif(results, 3))
```

|  | p.0 | p.1 | p.2 | mu.0 | mu.1 | mu.2 | sigma.0 | sigma.1 | sigma.2 |
| --- | --- | --- | --- | --- | --- | --- | --- | --- | --- |
| true | 0.90000 | NA | NA | 0.000000 | NA | NA | 1.0000 | NA | NA |
| mean (z) | 0.90000 | 0.05010 | 0.050400 | -0.000612 | 2.6000 | -2.5900 | 0.9870 | 1.9500 | 1.970 |
| stdev (z) | 0.00549 | 0.00423 | 0.004070 | 0.016100 | 0.0424 | 0.0426 | 0.0124 | 0.1650 | 0.165 |
| mean (z^2) | 0.90800 | 0.08060 | 0.011400 | 0.017400 | 2.6900 | -2.6900 | 0.9770 | 1.9900 | 1.360 |
| stdev (z^2) | 0.00500 | 0.00502 | 0.000435 | 0.015700 | 0.0556 | 0.0745 | 0.0119 | 0.0921 | 0.360 |

Interesting all effects are now positive but still the null is estimated quite well!!!

Back to top

# 5 Using simulated data cate

```
n <- 100  #number of samples
p <- 2000  #number of features
r <- 5  #number of unobserved covariates
nsim <- 100  #number of simulations
p0 <- 0.9  #proportion null
set.seed(12345)
sim.data <- simulate(n, p, r, nsim, p0)
table3 <- cbind(`Type I error` = summarize(sim.data[[1]]), Power = summarize(sim.data[[2]]))
rownames(table3) <- colnames(sim.data[[1]])
kable(table3)
```

|  | Type I error | Power |
| --- | --- | --- |
| naive | 0.72 (0.035) | 0.72 (0.046) |
| naive-gc | 0.0019 (0.0036) | 0.007 (0.0092) |
| naive-bc | 0.77 (0.05) | 0.78 (0.057) |
| cate | 0.061 (0.0061) | 0.85 (0.038) |
| cate.calibrate | 0.03 (0.0054) | 0.76 (0.053) |
| cate.bacon | 0.072 (0.0074) | 0.87 (0.039) |
| oracle | 0.053 (0.0056) | 0.84 (0.04) |

Back to top

# 6 The effect of different prior parameters

Using for the mean prior distribution a variance prior of 1/10 results in unstable Gibbs traces and elliptical posterior distribution.

```
set.seed(12345)
theta <- c(0.9, 0, 1, 1, 3, 1)
z <- rnormmix(p, theta)
bc <- bacon(z, priors = priors)
estimates(bc)
```

```
##           p.0        p.1        p.2          mu.0     mu.1      mu.2
## [1,] 0.863173 0.07846989 0.05835708 -0.0003810109 2.615812 -2.627495
##       sigma.0  sigma.1  sigma.2
## [1,] 0.940657 2.128642 1.605065
```

```
posteriors(bc)
```

```
traces(bc)
```

```
priors = list(sigma = list(alpha = 1.28, beta = 0.36), mu = list(lambda = c(0, 
    3, -3), tau = c(10, 10, 10)), epsilon = list(gamma = c(1, 1, 1)))
bc <- bacon(z, priors = priors)
posteriors(bc)
```

```
traces(bc)
```

```
estimates(bc)
```

```
##            p.0        p.1        p.2         mu.0     mu.1      mu.2
## [1,] 0.9196971 0.05231659 0.02798635 -0.006903698 2.782342 -2.832273
##        sigma.0  sigma.1  sigma.2
## [1,] 0.9835463 2.123704 1.442606
```

Back to top

# 7 Acknowledgement

This document is generated using *rmarkdown*/ *knitr* with the BioConductor-stylesheet from *BiocStyle*.

```
sessionInfo()
```

```
## R Under development (unstable) (2016-08-25 r71150)
## Platform: x86_64-pc-linux-gnu (64-bit)
## Running under: Ubuntu 14.04.5 LTS
## 
## locale:
##  [1] LC_CTYPE=en_US.UTF-8       LC_NUMERIC=C              
##  [3] LC_TIME=en_US.UTF-8        LC_COLLATE=en_US.UTF-8    
##  [5] LC_MONETARY=en_US.UTF-8    LC_MESSAGES=en_US.UTF-8   
##  [7] LC_PAPER=en_US.UTF-8       LC_NAME=C                 
##  [9] LC_ADDRESS=C               LC_TELEPHONE=C            
## [11] LC_MEASUREMENT=en_US.UTF-8 LC_IDENTIFICATION=C       
## 
## attached base packages:
## [1] parallel  stats     graphics  grDevices utils     datasets  methods  
## [8] base     
## 
## other attached packages:
##  [1] RColorBrewer_1.1-2        knitr_1.14               
##  [3] matrixStats_0.51.0        edgeR_3.16.1             
##  [5] limma_3.28.20             tweeDEseqCountData_1.12.0
##  [7] Biobase_2.34.0            BiocGenerics_0.20.0      
##  [9] locfdr_1.1-8              bacon_1.2.0              
## [11] ellipse_0.3-8             BiocParallel_1.8.0       
## [13] ggplot2_2.1.0             BiocStyle_2.2.0          
## 
## loaded via a namespace (and not attached):
##  [1] Rcpp_0.12.7      magrittr_1.5     splines_3.4.0    munsell_0.4.3   
##  [5] lattice_0.20-33  colorspace_1.2-7 stringr_1.1.0    plyr_1.8.4      
##  [9] tools_3.4.0      grid_3.4.0       gtable_0.2.0     htmltools_0.3.5 
## [13] yaml_2.1.13      digest_0.6.10    formatR_1.4      evaluate_0.10   
## [17] rmarkdown_1.0    stringi_1.1.2    scales_0.4.0     locfit_1.5-9.1
```

Back to top

# References

Devlin, B, and K Roeder. 1999. “Genomic control for association studies.” *Biometrics* 55 (4): 997–1004.

Efron, B. 2004. “Large-Scale Simultaneous Hypothesis Testing: The Choice of a Null Hypothesis.” *JASA* 99 (465).

Efron, B, and T Hastie. 2016. “Large-Scale Hypothesis Testing and FDRs.” In *Computer Age Statistical Inference*, edited by Holmes Cox Hambly, 271–94. New York: Cambrige university press.

Efron, B. 2010. “Correlation Questions.” In *Large-Scale Inference*, edited by Holmes Cox Hambly, 141–62. New York: Cambrige university press.

Wang, J, Q Zhao, T Hastie, and A B Owen. 2015. “Confounder Adjustment in Multiple Hypothesis Testing.” *ArXiv E-Prints*. http://arxiv.org/abs/1508.04178.
